# Inversion of the Chiroptical Responses of Chiral Gold Nanoparticles with a Gold Film

**DOI:** 10.1021/acsnano.3c07475

**Published:** 2023-12-21

**Authors:** Yilin Chen, Jiapeng Zheng, Lingling Zhang, Shasha Li, Yang Chen, Ka Kit Chui, Wei Zhang, Lei Shao, Jianfang Wang

**Affiliations:** † Department of Physics, The Chinese University of Hong Kong, Shatin, Hong Kong SAR 999077, China; ‡ Institute of Applied Physics and Computational Mathematics, Beijing 100088, China; § State Key Laboratory of Optoelectronic Materials and Technologies, Guangdong Province Key Laboratory of Display Material and Technology, School of Electronics and Information Technology, Sun Yat-sen University, Guangzhou 510275, China

**Keywords:** chiroptical responses, chiral plasmonic nanoparticles, circular differential scattering, gold film, gold nanoparticles, plasmon resonance

## Abstract

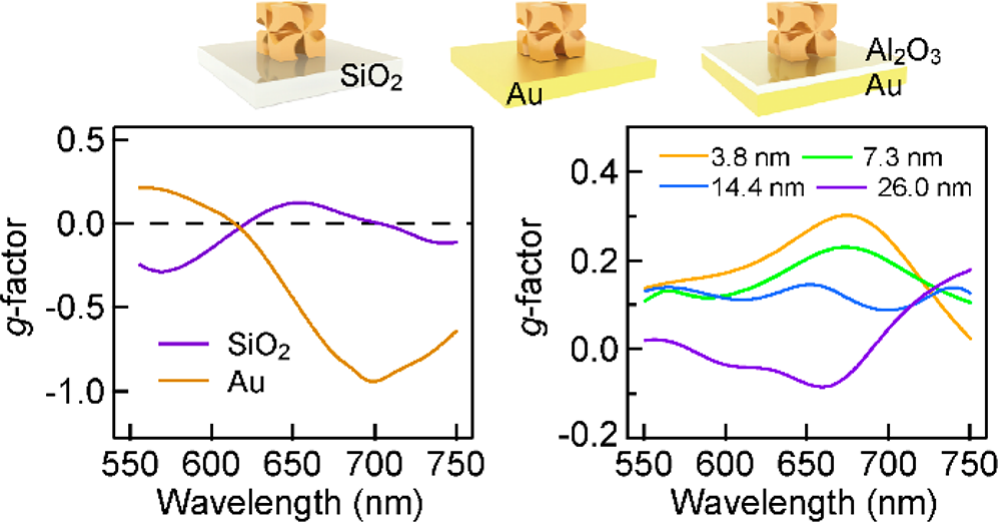

The deposition of chiral nanoparticles (NPs) onto various substrates is crucial for the fabrication of high-density photonic devices. Understanding the interaction of chiral light and chiral NPs supported on substrates is essential for developing optical sensors and modulators. However, the chiroptical responses of plasmonic chiral NPs on substrates have remained elusive. Here we provide an important understanding of the correlation between the substrate material and the chiroptical response. The scattering dissymmetry factors of individual chiral Au nanocubes are inverted and enhanced with a gold film. Qualitative theories are proposed to analyze the observed variations in the chiroptical signals of chiral NPs on different substrates. Our results offer an encouraging route for modulating and amplifying the chiroptical signals in the use of chiral NPs in light control, light-based quantum technologies, and sensing.

Chiral matter, whose inherent and mirror images cannot be superimposed, interacts differently with left- and right-handed circularly polarized (LCP and RCP) light. Plasmonic nanoparticles (NPs), such as nanorods and nanocubes, modified by chiral ligands, can provide strong near-field to give detectable chiroptical responses.^1^ However, the coupling between the localized surface plasmon resonance and the dipoles of chiral molecules is relatively weak. Assembly of plasmonic NPs into plasmonic nanostructures with chiral geometries has been employed for the amplification of chiroptical signals. It can allow multiple hotspots to be excited and thus significantly promote light–matter interaction, leading to larger far-field dissymmetry factors (*g*-factors).^2^ Great efforts have been devoted to the assembly of chiral nanostructures from achiral ingredients by introducing molecular linkers and soft templates in solution.^2^ However, soft frameworks tend to deform the geometrical configurations of the assembled chiral structures after being dried. The fragility and large footprints make it difficult for the chiral assemblies to be further integrated with other materials to create functional structures and devices. Wet-chemistry synthesis is a competitive and promising strategy to address such challenges. Advances in the colloidal synthesis of metal NPs have enabled the formation of distinct chiral features on the individual NPs.^3−7^ Free electrons on the surface of a chiral metal NP move along a helical trajectory under the excitation of circularly polarized light (CPL).^8^ Chiral plasmonic NPs therefore possess nonorthogonal electric and magnetic dipole moments, which result in pronounced optical chiralities.^9^ Compared to chiral assemblies out of achiral NPs, individual chiral NPs have the advantages of good stability, high yields, and extremely small footprints. Chiral NPs with proper pitches and sharp protrusions enable the generation of an exotic chiral current and tightly localized electromagnetic enhancement. They therefore not only exhibit distinctly different responses to LCP and RCP light but also support strong enhancement of near-field optical chirality.^10^

Miniaturized optical components with giant chirality have stimulated intense interest in the manipulation of light polarization,^11^ ultrasensitive localized spectral detection,^12^ and quantum optical computing.^13^ Such applications require flexible integration of chiral components with other functional components to create functional materials and devices. Chiral plasmonic metal NPs offer an important opportunity for this purpose. However, plasmonic metal NPs are generally required to be deposited on various substrates. This requirement is also applicable for chiral metal NPs.^14^ For example, chiral NPs have been deposited on quartz substrates to study their linear and nonlinear chiroptical responses.^15^ Chiral NPs have also been utilized for circularly polarized luminescence on metal, semiconductor, and insulator substrates, which can be further employed to fabricate optoelectronic devices, such as lasers and light-emitting diodes.^16−18^ The introduction of a substrate leads to the symmetry breaking of the dielectric environment surrounding chiral metal NPs. Such a break in symmetry has a significant impact on the chiroptical response. Prior studies have produced much evidence on the control over the polarization properties of transmitted and reflected light based on the variation of substrates.^12,19^ However, predicting the chiroptical responses of chiral NPs has remained challenging. Studying the effect of different supporting substrates on the chiroptical properties of individual chiral NPs is therefore of great importance. The fundamental understanding underlies the essential physics of various plasmonic devices. The local electromagnetic field can be strongly enhanced in the gap between NPs and a metal film.^20^ A small change in the properties of the material in the gap results in a large change in the plasmon resonance. The potential applications of the NP-on-mirror structure have been intensively cultivated in the recent years, including plasmon-enhanced fluorescence, strong coupling, nonlinear optical signals, and surface-enhanced Raman scattering.^21^ In addition, metal substrates with high reflectivities have been proposed for amplifying the chiroptical signals of supported chiral molecules owing to the superchiral electromagnetic field generated by the interference between the incident CPL and the reflected light above the metal substrate.^3,22^ In this regard, the construction of (chiral nanoparticle)-on-mirror structures by depositing chiral Au NPs on a gold film is highly desirable; yet, this significant scenario has remained unexplored.

In this work, we studied the correlation between substrate materials and the chiroptical responses of chiral gold nanocubes (CGNCs). Single-particle circular differential scattering was performed to investigate the chiroptical responses of the individual CGNCs supported by different substrates under the excitation of CPL. The difficulties of the signal fluctuation arising from slight variations in the morphology of the CGNCs were avoided by transferring the CGNCs from silica substrates onto gold films. The localized electromagnetic field is significantly enhanced when a CGNC is separated from a gold film with a thin dielectric layer, which results in a large differential scattering. The scattering *g*-factor is inverted and enhanced when the substrate material is changed from SiO_2_ to Au. Averaging over the D- and L-handed CGNCs with random orientations on Au films also shows good mirror symmetry. These experimental observations are well understood through the simulations of the scattering spectra, electromagnetic field distributions, and optical chirality distributions. The chiroptical response was further varied by changing the thickness of the dielectric layer in the gap between the CGNC and the Au film. The chiroptical response of the CGNC-on-substrate structure is not only derived from its highly twisted surface feature but also highly dependent on the dielectric properties of the substrate. A qualitative theory was proposed to analyze the effect of the substrate on the chiroptical response of the CGNC. The CGNC-on-mirror (CGNCoM) structure is a promising platform for manipulating and amplifying the chiroptical signals of chiral NPs. Our results of the substrate-based optical chirality in nanoscale chiral systems offer a perspective on chiral light–matter interaction at substrate–nanostructure interfaces.

## Results and Discussion

**Figure 1:**
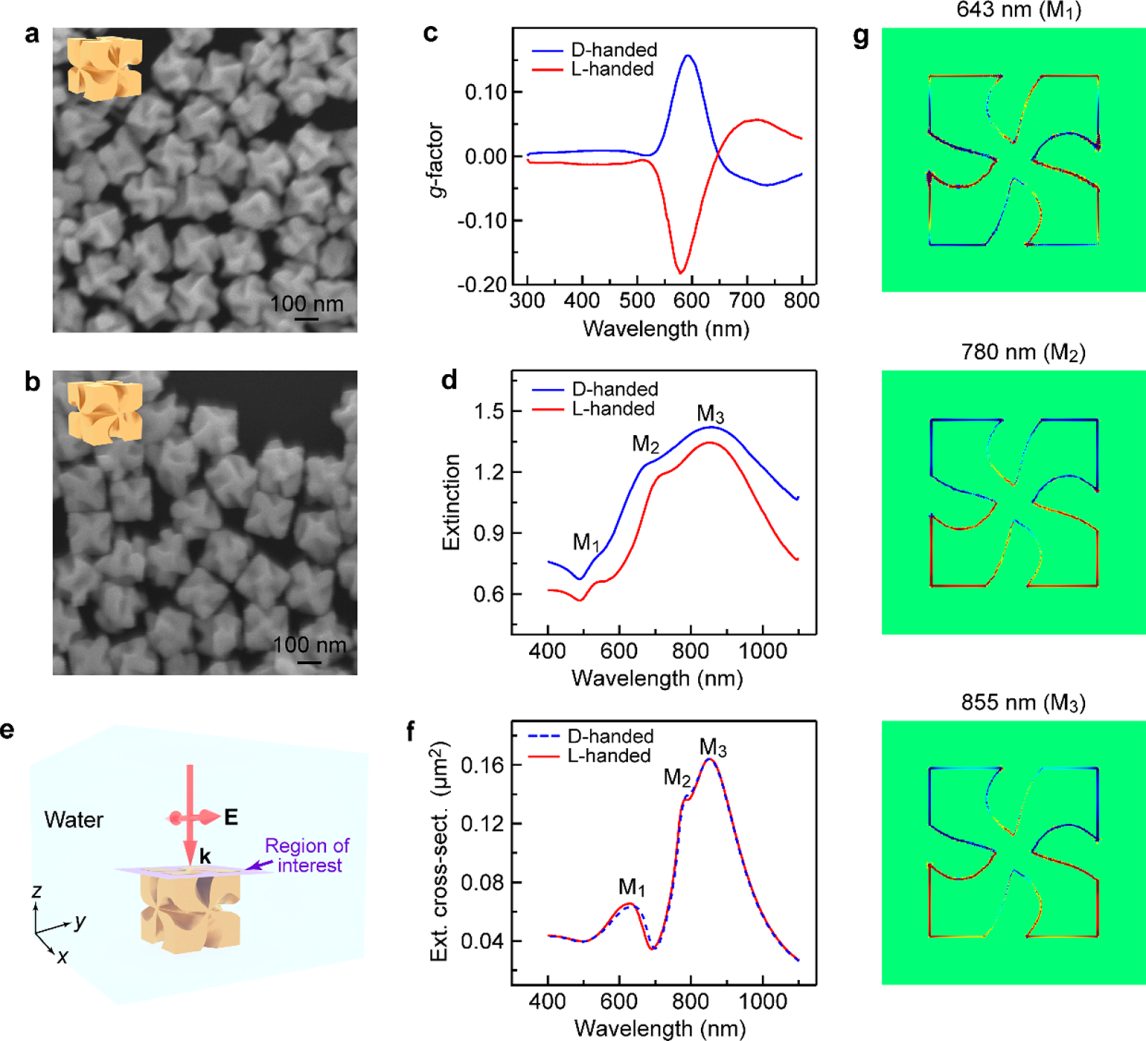
Synthesis and plasmonic properties of the D- and L-handed CGNCs. (a,b) Scanning electron microscopy (SEM) images of the D- (a) and L-handed (b) CGNCs with distinct handedness. (c,d) Extinction *g*-factor (c) and extinction (d) spectra of the CGNC samples. (e) Schematic of the three-dimensional model of a CGNC immersed in water (*n* = 1.33) for the simulations. (f) Simulated extinction cross-section spectra of the D- and L-handed CGNCs. (g) Simulated charge distribution contours in the plane crossing the CGNC as given by the purple square in (e). Red: positive charges; blue: negative charges.

The D- and L-handed CGNCs with an edge length *L* of ∼160 nm were synthesized through overgrowth on Au nano-octahedra in solutions containing glutathione (GSH) molecules with chiral conformations (see Methods). The GSH molecules tethered on the Au surface were used to control the chiral growth on the high-Miller-index facets. D- and L-GSH finally led to the formation of D- (Figure 1a) and L-handed CGNCs (Figure 1b), respectively. The twisty arms of the CGNCs are connected in a fourfold rotational symmetry and produce a chiral structure (Figure S1). Their highly twisted surface feature and high crystallinity are prominent in the following spectroscopic studies. The circular dichroism (CD) spectrum of the aqueous D-handed CGNC sample shows an ensemble asymmetry factor of *g*_e_ = 0.18 (Figure 1c). The extinction *g*-factor is defined as *g*_e_ = 2 × (*E*_LCP_ – *E*_RCP_)/(*E*_LCP_ + *E*_RCP_), where *E*_LCP_ and *E*_RCP_ are the extinction values of the aqueous CGNC sample under the excitation of LCP and RCP light, respectively. The extinction *g*-factors of the D- and L-handed CGNCs show opposite chirality in the wavelength range of 500–800 nm. The extinction spectrum of the L-handed CGNCs shows three plasmon resonance peaks at 537, 716, and 852 nm, which are defined as plasmon resonance modes M_1_, M_2_, and M_3_, respectively (Figure 1d). Take the L-handed CGNCs as an example. The negative *g*-factor band at 578 nm is caused by the resonance modes M_1_ and M_2_, while the positive *g*-factor band at 716 nm is caused by the resonance modes M_2_ and M_3_. The plasmonic properties of a CGNC embedded in a homogeneous medium were analyzed by the finite-difference time-domain (FDTD) method (Figure 1e, see also Methods). The simulated extinction cross-sectional spectrum of the CGNC in Figure 1f shows three distinct plasmon resonance modes, which correspond to the quadrupole mode (M_1_) at 643 nm, dipole mode (M_2_) at 780 nm, and dipole mode (M_3_) at 855 nm. The distributions of charges in the CGNC at the three peak wavelengths are shown in Figure 1g.

**Figure 2:**
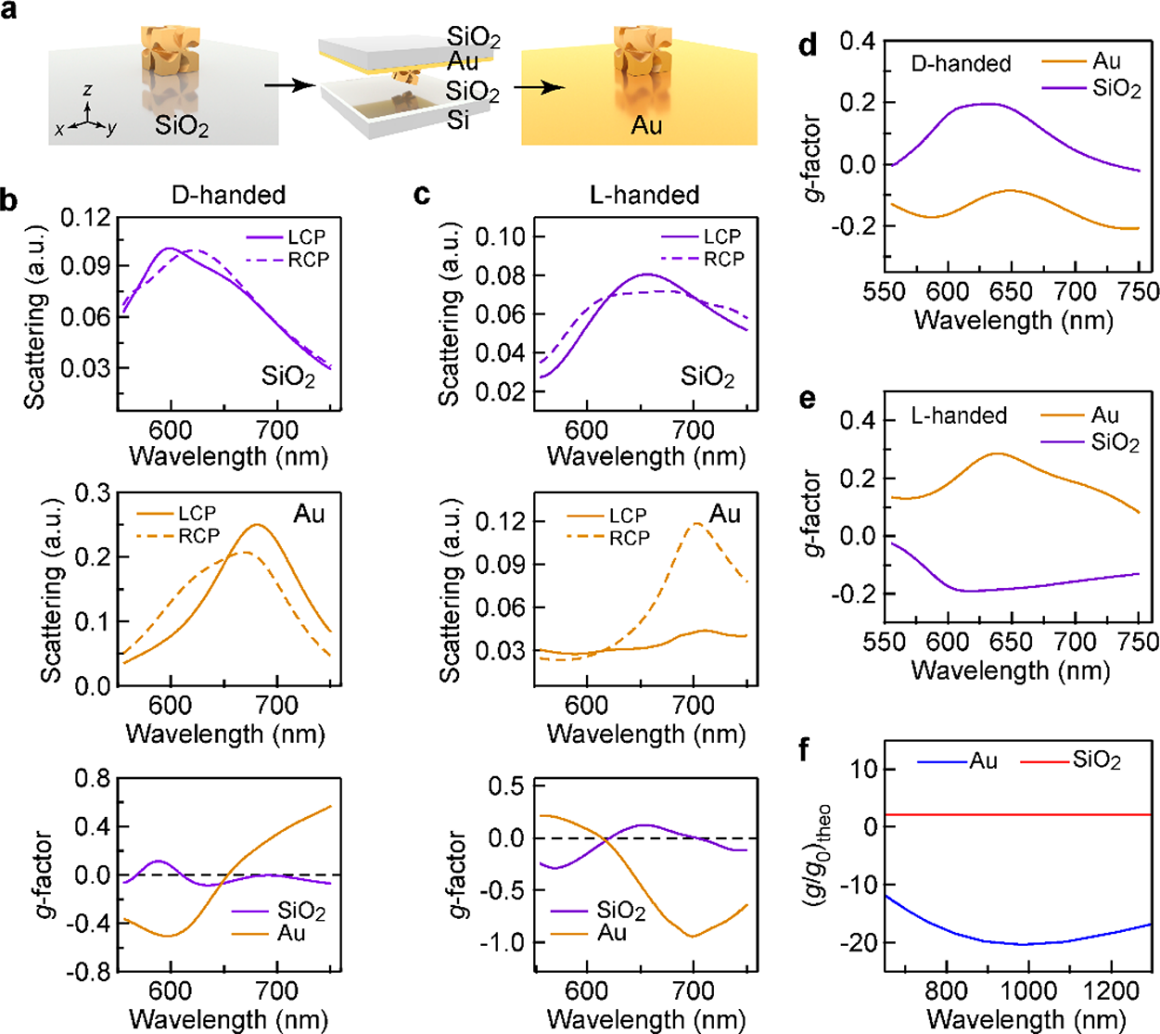
Circular differential scatterometry. (a) Schematic showing the transfer process of an individual CGNC from a silica substrate to a gold film. (b) Scattering spectra under the excitation of CPL and scattering *g*-factor spectra of an individual D-handed CGNC supported on silica (purple lines) and a gold substrate (golden lines). (c) Scattering spectra and scattering *g*-factor spectra of an L-handed CGNC. The solid and dashed lines represent the scattering spectra under the excitation of LCP and RCP light, respectively. The horizontal dashed black lines represent the zero lines of the *g*-factor. (d) Average scattering *g*-factor spectra of the D-handed CGNCs supported on SiO_2_ (purple line) and Au (golden line) substrates. (e) Average scattering *g*-factor spectra of the L-handed CGNCs. (f) Calculated (*g*/*g*_0_)_theo_ spectra. The blue and red lines represent the (*g*/*g*_0_)_theo_ spectra for the Au and SiO_2_ substrates, respectively.

Circular differential scattering can be an important part of the CD response because the CGNCs possess large scattering cross-sections (Figure S2). Dark-field differential scatterometry was used to characterize the chiroptical signals of the individual CGNCs (Figure S3a,b, see also Methods). The backward scattering *g*-factor is defined as *g*_s_ = 2 × (*S*_LCP_ – *S*_RCP_)/(*S*_LCP_ + *S*_RCP_), where *S*_LCP_ and *S*_RCP_ are the scattering signals under the excitation of LCP and RCP light, respectively. The dark-field scattering image (Figure S3c) shows that the as-prepared CGNCs were sparsely deposited on substrates. However, slight variations in the morphologies of the CGNCs result in pronounced chiroptical differences. Such a signal fluctuation hindered the correlation of the geometrical chirality of the CGNCs to their chiroptical properties. The same CGNCs were therefore transferred and probed (see Methods). The scattering *g*-factors of the CGNCs supported on a SiO_2_ substrate were first measured. The targeted CGNCs on the SiO_2_ substrate were then transferred onto a gold film for further optical measurements (Figure 2a). The same CGNCs were probed and compared on the two types of the substrates. Such a process eliminates the effect of the slight difference in the morphology of the CGNCs on the scattering *g*-factor. The scattering *g*-factors of the CGNCs on Au films can then be reliably compared with those of the same CGNCs supported on SiO_2_ substrates. The chiral spectral features of the D- and L-handed CGNCs are presented in the spectral range of 550–750 nm (Figure 2b,c). The scattering intensity of the individual CGNCs separated from the Au film by a cetyltrimethylammonium bromide (CTAB) bilayer is enhanced due to the localized electromagnetic field enhancement. The distribution of the far-field radiation of the CGNC on a Au film is vastly different from that of the CGNC in a homogeneous medium or on a dielectric substrate. The radiation intensity of the CGNC on a Au film is stronger compared to that in water or on a SiO_2_ substrate. The differential scattering (Δ*S*) of the Au-supported CGNCs is therefore larger than that of the SiO_2_-supported CGNCs. Δ*S* is defined as the difference between *S*_LCP_ and *S*_RCP_. When a D-handed CGNC is transferred from the SiO_2_ substrate to the Au film, the scattering peak is red-shifted to 682 nm and increased in intensity under the excitation of LCP light. The absolute value of the scattering *g*-factor peak for the Au-supported D-handed CGNC at 597 nm is ∼4.47 times that of the peak for the same CGNC supported on a SiO_2_ substrate at 588 nm. In contrast, the scattering peak in the spectrum of an L-handed CGNC supported on a gold film is red-shifted to 703 nm with an enhanced intensity under the excitation of RCP light. Circular differential scatterometry on the randomly picked D- and L-handed CGNCs based on single-particle transfer was additionally carried out (Figures S4 and S5). The scattering spectra of the CGNCs show varying numbers and different spectral positions of the plasmon resonance peaks under the excitation of CPL. The scattering *g*-factors of the CGNCs are also inverted when the substrate material is changed from SiO_2_ to Au.

Circular differential scatterometry can not only give the chiroptical responses of individual chiral NPs but also reveal the chiral features of chiral NPs through averaging of the scattering *g*-factor spectra. Large differences in the number and positions of the peaks or dips in the scattering *g*-factor spectra were observed (Figure S6). Only a single disperse feature remains in the average spectrum, while the minor spectral signatures vanish (Figure 2d,e). Each average *g*-factor spectrum was calculated from 25 CGNCs. A peak in the average *g*-factor spectrum for the D-handed CGNCs supported on SiO_2_ substrates appears at 631 nm (Figure 2d), while a dip in the average *g*-factor spectrum for the SiO_2_-supported L-handed CGNCs appears at 617 nm (Figure 2e). In contrast, two dips in the average *g*-factor spectrum for the D-handed CGNCs supported on Au films appear at 586 and 742 nm, while a peak in the average *g*-factor spectrum for the Au-supported L-handed CGNCs appears at 639 nm. The blue-shift of the average *g*-factor band can be ascribed to the smaller sizes of the picked L-handed CGNCs. The average spectra of the scattering *g*-factors of the CGNCs supported on SiO_2_ substrates and on Au films therefore reproduce the inversion of the chiroptical response.

The chiroptical response of a chiral NP supported on a substrate was analyzed qualitatively. A chiral NP subjected to a monochromatic electromagnetic field generates an electric dipole (ED) moment  and a magnetic dipole (MD) moment .^22,23^ The chiroptical response of a chiral NP is determined by the rotational strength *S* = Im( · ),where Im stands for the imaginary part of the complex parameter in the parentheses.^24^ can be decomposed into  and , with the subscripts ∥ and ⊥ representing the dipole moment components oriented parallel and perpendicular to the substrate, respectively. In an NP-on-mirror structure,  gives rise to an antiparallel mirror ED moment , while  gives rise to a parallel mirror MD moment .^25^ For the case of a substrate with a dielectric function of ,  can be written as , where *K* = (1 – )/( + 1).  can be expressed as . The rotational strength of the image charges *S*′ in the substrate can be derived from –Im( · ). *S*′ for a chiral NP supported by a perfect mirror equals the opposite of the original *S* of the chiral NP itself, which fits with the concept that the mirror image of a D-handed NP is L-handed. The generated dipole moments by an NP on a substrate can therefore be described by an effective ED moment  and MD moment , with contributions from both the NP and the image NP. They can be written as
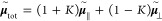
1
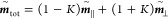
2

Therefore, the *g*-factor of a chiral NP supported by a substrate can be calculated according to
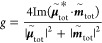
3*g*_0_ is the *g*-factor of the chiral NP in vacuum. It can be expressed as
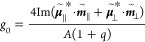
4where *q* = *B*/*A*, with *A* = ||^2^ + ||^2^ and *B* = ||^2^ + ||^2^. We define a ratio of
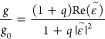
5to represent the effect of the dielectric function of the substrate on the chiroptical response of a chiral nanostructure constructed by a chiral NP and its supporting substrate. Re stands for the real part of the complex parameter in the parentheses. The above formula depicts that the *g*-factor of the chiral nanostructure changes its sign with Re(). See the Methods for the theory for the contribution of the electric quadrupole (EQ) mode to the chiroptical response. The EQ moment plays a role similar to that of the MD moment for the chiroptical response in the presence of a Au substrate. When the contributions from both the MD and EQ moments are taken into account, the overall *g*/*g*_0_ still has the form of eq 5 except for the modification of the factor *q* based on the components of the ED, MD, and EQ moments in the chiral NP. Figure 2f shows the ratios of (*g*/*g*_0_)_theo_ for chiral NPs supported on SiO_2_ and Au substrates, respectively, as functions of wavelength. In the calculation, *q* = 0.0006. (*g*/*g*_0_)_theo_ for SiO_2_ substrates in the spectral range of 400–900 nm is ∼2.1. When the substrate material is changed from SiO_2_ to Au, (*g*/*g*_0_)_theo_ becomes negative in the spectral range of 650–1200 nm, and the minimum value is −20 at the wavelength of 984 nm. The absolute value of the *g*-factor for Au substrates at 597 nm is ∼4.1 times that for SiO_2_ substrates.

The chiroptical responses of the three-dimensional CGNC models were simulated using FDTD under the excitation of normally incident CPL to compare with the theoretical results. Figure S7a shows the wavelength-dependent ratios of *g*_silica_/*g*_0_ and *g*_gold_/*g*_0_. The *g*_silica_/*g*_0_ spectrum exhibits positive values of ∼4.49 in the wavelength range of 777–1200 nm. *g*_gold_, *g*_silica_, and *g*_0_ represent the scattering *g*-factor spectra of the CGNC on a gold substrate, on a silica substrate, and under vacuum, respectively. In contrast, a dip value of *g*_gold_/*g*_0_ reaches −27.2 at a wavelength of 992 nm. The signs of the scattering *g*-factors of the CGNC are inverted in the wavelength range of 657–1200 nm when the substrate material is changed from SiO_2_ to Au. Similar FDTD simulations have also been conducted to analyze the chiroptical responses of the L-handed CGNC on different substrates. The scattering *g*-factor spectra of the L-handed CGNC exhibit opposite chiroptical responses when compared to the results of the D-handed CGNC. The spectra of *g*_silica_/*g*_0_ and *g*_gold_/*g*_0_ for the L-handed CGNC shown in Figure S7b demonstrate similar results to those depicted in Figure S7a for the D-handed CGNC. These spectra of *g*_gold_/*g*_0_ and *g*_silica_/*g*_0_ obtained from FDTD can be compared with the (*g*/*g*_0_)_theo_ in Figure 2f calculated using eq 5. It is important to note that the accuracy of the theoretical results in Figure 2f is valid only when the factor *q* is considered constant at 0.0006 within the wavelength range. The value of the factor *q* can vary significantly with wavelength, especially when strong plasmon resonance occurs. In such cases, the evaluation of (*g*/*g*_0_)_theo_ should take into account the specific values of the factor *q*.

**Figure 3:**
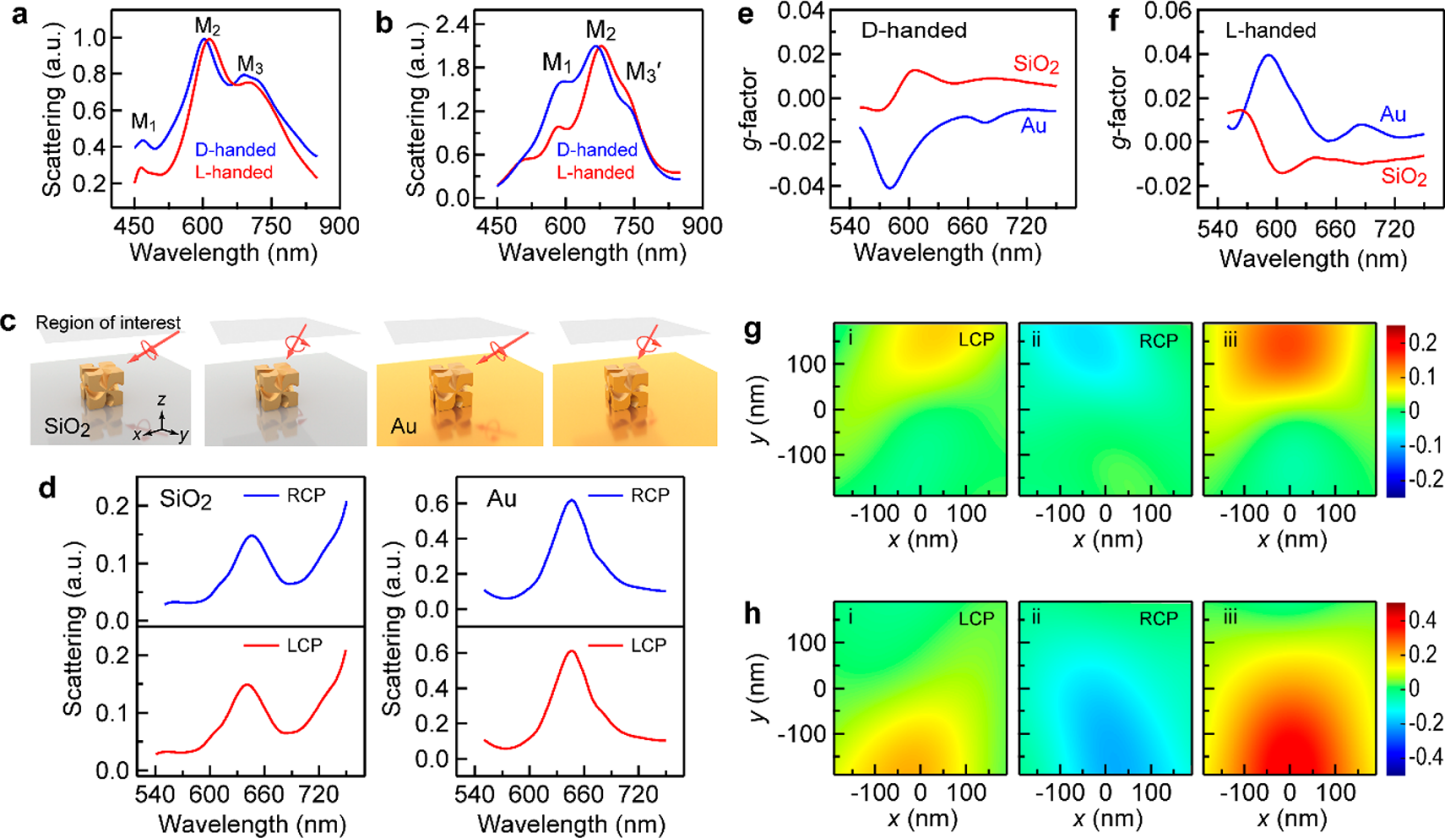
Plasmon resonance modes and chiroptical response of the CGNC. (a,b) Scattering spectra of the CGNC supported on SiO_2_ (a) and Au (b) substrates. The blue and red lines represent the spectra of the D- and L-handed CGNC, respectively. (c) Schematics of the CGNC under the excitation of LCP light. The excitation light is incident toward the edge and corner, respectively. The planes above the CGNC represent the regions for the simulation of the scattering power and electromagnetic field. (d) Simulated scattering spectra of the CGNC supported on SiO_2_ and Au substrates, respectively. The blue and red lines represent the scattering spectra under the excitation of RCP and LCP light, respectively. (e,f) Simulated scattering *g*-factor spectra of the D- (e) and L-handed (f) CGNC. The red and blue lines correspond to the scattering *g*-factor spectra of the CGNC supported on SiO_2_ and Au substrates, respectively. (g,h) Contours of the optical chirality enhancement *C*_LCP_/|*C*_0_| (i), *C*_RCP_/|*C*_0_| (ii), and (*C*_LCP_ – *C*_RCP_)/|*C*_0_| (iii) in the regions of interest above the CGNC supported on SiO_2_ (g) and Au (h) substrates. The red and blue colors represent positive and negative quantities of *C*, respectively.

We studied the plasmon resonance and chiroptical response of the CGNCs supported on different substrates. Dark-field scattering spectra under the excitation of unpolarized light were measured. Three plasmon resonance peaks were observed at 464, 613, and 701 nm in the scattering spectrum of the SiO_2_-supported L-handed CGNC (Figure 3a). The three peaks are blue-shifted in comparison with those for the aqueous L-handed CGNC sample because of the reduction in the overall refractive index of the surrounding environment. FDTD was employed to simulate the scattering spectra of the 3D CGNC model. All simulations were performed on the individual CGNCs placed on substrates and embedded in the surrounding medium of *n* = 1.0. Linearly polarized light was illuminated on the substrates obliquely to simulate the dark-field excitation configuration. The simulated scattering spectra for the SiO_2_-supported CGNC show that the resonance modes induced by s- and p-polarized excitation both contribute to the scattering (Figure S8). The scattering spectrum for the Au-supported L-handed CGNC shows a quadrupole resonance at 583 nm (M_1_) and a dipole resonance at 677 nm (M_2_) (Figure 3b). The simulated scattering spectra (Figure S9) reveal that M_1_ and M_2_ are mainly excited by s-polarized light, while M_3_′ is excited by p-polarized light. To better understand the resonance modes of CGNC-on-Au, we calculated the contours of the electric field and charges in one *x*-*y* plane crossing the bottom surface of the CGNC and one *x*-*y* plane placed at the surface of the Au film (Figure S10). For M_1_ at 550 nm, the induced image dipole resonance in the Au film cancels the original dipole resonance. The overall scattering intensity of in-plane mode M_1_ is therefore attenuated. For M_2_ at 661 nm, the enhancement of the electric field in the Au film is significantly reduced. Such weak image dipole resonance is not enough to cancel the original one, resulting in a scattering peak at 661 nm.

To ascertain the inversion of the chiroptical response, we simulated the scattering *g*-factor spectra by FDTD and further calculated the contours of the optical chirality. The scattering spectra were averaged over incident directions with eight azimuth angles φ varied from 0 to 315° at a step of 45° and a fixed polar angle θ of 64°. Figure 3c shows the cases of CPL illuminating at the edge (i.e., φ = 0°) and the corner (i.e., φ = 45°) of the CGNC supported on SiO_2_ and Au substrates. Take the D-handed CGNC as an example. The scattering spectra of CGNC-on-SiO_2_ and CGNC-on-Au under the excitation of LCP and RCP light were simulated (Figure 3d). The increase in the simulated scattering power in the near-infrared region for the CGNCs on the SiO_2_ substrate is attributed to the strong dipolar mode of M_3_ (Figures 3a and S8). The scattering *g*-factor spectra were then calculated from the average scattering spectra using the expression of *g*_s_ (Figure 3e). Two positive bands of the *g*-factor for CGNC-on-SiO_2_ appear in the range of 580–750 nm, while two negative bands for CGNC-on-Au appear in 550–750 nm. The simulated *g*-factor spectra of the L- and D-handed CGNC supported on substrates with the same properties show chiral features with good mirror symmetry (Figure 3e,f). The simulation proves that the two *g*-factor bands in the wavelength range of 550–750 nm are inverted when the supporting substrate is changed from SiO_2_ to Au. Moreover, the degree of the chiral asymmetry of the electromagnetic field in the region of interest above the CGNC was determined by the optical chirality *C*.^22^ The optical chirality enhancements *C*/|*C*_0_| under the excitation of 661 nm CPL are presented in Figure 3g,h. *C*_0_ is the optical chirality of CPL in vacuum. Take the D-handed CGNC as an example (Figure 3g). The contours of the optical chirality enhancements *C*_LCP_/|*C*_0_| and *C*_RCP_/|*C*_0_| show opposite signs in the region above a SiO_2_-supported CGNC. The subscripts LCP and RCP indicate that *C* was calculated under the excitation of LCP and RCP light, respectively. As the substrate material is changed from SiO_2_ to Au (Figure 3h), the contours of *C*_LCP_/|*C*_0_|, *C*_RCP_/|*C*_0_|, and differential optical chirality enhancements (*C*_LCP_ – *C*_RCP_)/|*C*_0_| in the region of interest are inverted in sign and increase in absolute ratio.

**Figure 4:**
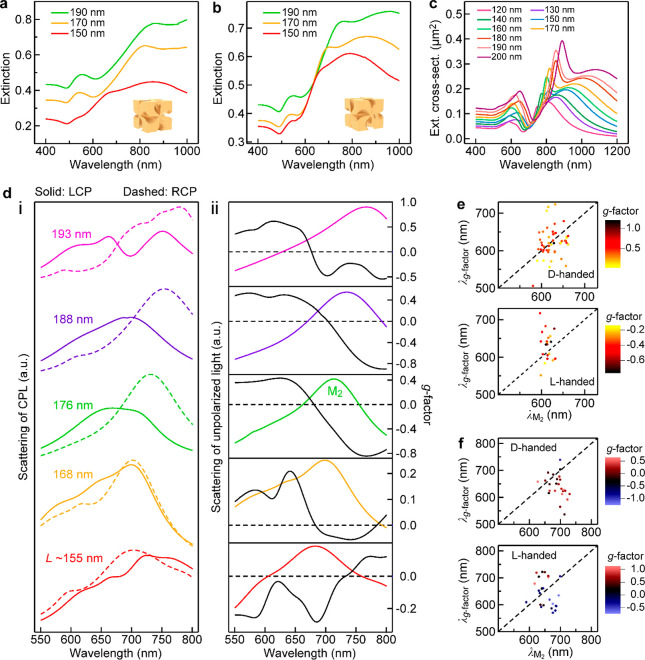
Scattering *g*-factors and plasmon resonance. (a,b) Extinction spectra of the aqueous D- (a) and L-handed (b) CGNC samples. (c) Simulated extinction cross-section spectra of the CGNCs immersed in water. (d) Scattering and *g*-factor spectra of the Au-supported D-handed CGNCs with different *L* values. The scattering spectra in (i) were measured under the excitation of CPL. The solid and dashed lines represent the scattering spectra under the excitation of LCP and RCP light, respectively. The solid black curves in (ii) represent the corresponding scattering *g*-factor spectra. The red, yellow, green, purple, and pink lines in (ii) represent the scattering spectra of the corresponding CGNCs under the excitation of unpolarized light. The dashed black lines represent the zero lines of the *g*-factor. (e) Wavelengths of the M_2_ mode and *g*-factor band for the D- and L-handed CGNCs supported on SiO_2_ substrates. The dashed black lines represent the diagonal lines. (f) Wavelengths of the M_2_ mode and *g*-factor bands for the CGNCs supported on Au films. The wavelengths of the distinct peaks in the *g*-factor spectra with positive values marked in red color. The wavelengths of the distinct dips with negative *g*-factor values marked in blue color.

We further analyzed the correlation between the chiroptical response and the plasmon resonance. The D- and L-handed CGNCs with different average lengths *L* were synthesized, as shown with the SEM images (Figure S11). The extinction spectra (Figure 4a,b) show that the quadrupole resonance mode M_1_ is slightly shifted, while the dipole resonance modes M_2_ and M_3_ are red-shifted with the increase of *L*. Such a spectral variation with the CGNC size fits well with the simulated extinction and scattering cross-sectional spectra of the CGNCs with different *L* values (Figures 4c and S12). The plasmon resonance modes can therefore be adjusted by the CGNC size. To explore the correlation between the single-particle chiroptical response and the plasmon resonance, scattering and *g*-factor spectra were measured on the individual SiO_2_-supported D- and L-handed CGNCs with different *L* values, respectively (Figures S13 and S14). The plasmon resonance peak wavelengths of M_2_ (M_2_ wavelength) and *g*-factor bands are red-shifted slightly as *L* is increased. The scattering spectra and *g*-factor spectra of the Au-supported D- and L-handed CGNCs are presented in Figures 4d and S15, respectively. Take Figure 4d is an example. The plasmon resonance under the excitation of RCP light exhibits significant red-shifts as *L* is increased from 155 to 193 nm. Multipole resonance modes appear when *L* is 193 nm. When the M_2_ wavelength is 712 nm as shown with the green lines, the dip in the *g*-factor band is red-shifted to 768 nm, which is beyond the detection limit of our chiroptical detection system (maximum wavelength = 750 nm). A *g*-factor peak of ∼0.6 at 613 nm was measured from a CGNC instrument with *L* being 193 nm. Such a variation of the *g*-factor with the M_2_ wavelength shows the correlation between the plasmon resonance and the chiroptical response. To ascertain such a correlation, more than 40 CGNCs on SiO_2_ and Au substrates were measured, and the wavelengths of the plasmon and chiral resonance peaks were plotted in Figure 4e,f, where the *x*-axis represents the M_2_ wavelength and the *y*-axis represents the wavelength of the peak or dip of the *g*-factor band. Compared to the M_2_ wavelengths, the wavelengths of the *g*-factor peaks and dips are distributed in a broader range. The *g*-factor peaks or dips of CGNC-on-SiO_2_ positioned within ∼50 nm around their M_2_ wavelengths are more likely to give higher degrees of *g*-factor. For the Au-supported D-handed CGNCs, the *g*-factor peak wavelengths in 650–750 nm are longer than their M_2_ wavelengths. As the M_2_ wavelength is increased to the range of 700–750 nm, the wavelength of the *g*-factor dip in 550–620 nm is shorter than the M_2_ wavelength. Such phenomena were also observed in the *g*-factors for Au-supported L-handed CGNCs. These results reveal that the values and positions of the *g*-factor peaks and dips are highly dependent on the plasmon resonance. Such a correlation is important for predicting the chiroptical response of chiral plasmonic NPs.

**Figure 5:**
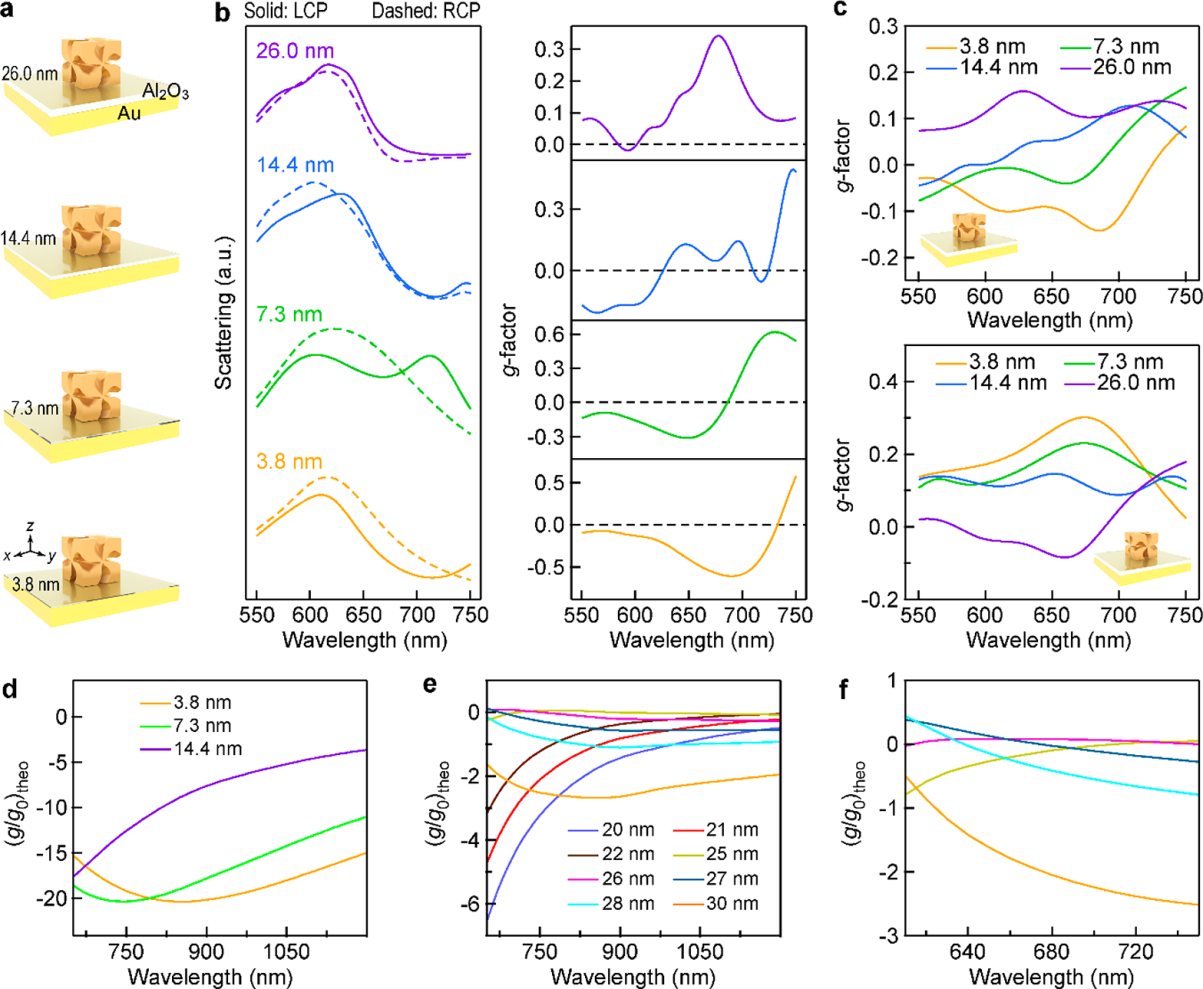
Dependence of the chiroptical response on the spacer thickness. (a) Schematics of the CGNCs supported on the Au/Al_2_O_3_ bilayer substrates. (b) Scattering spectra under the excitation of CPL and *g*-factor spectra of the D-handed CGNCs supported on the bilayer substrates with different Al_2_O_3_ layer thicknesses. (c) Average *g*-factor spectra of the D- (upper) and L-handed (lower) CGNCs. (d,e) (*g*/*g*_0_)_theo_ spectra for the chiral NPs supported on the bilayer substrates. The thickness of the Al_2_O_3_ layer is varied from 3.8 to 14.4 nm (d) and from 20 to 30 nm (e). (f) Partial view of the (*g*/*g*_0_)_theo_ spectra in (e) in the wavelength range of 610–750 nm. The thickness of the Al_2_O_3_ layer is varied from 25 to 30 nm.

To further ascertain the effect of Au films on the chiroptical response, the CGNCs were deposited on Au films coated by a dielectric layer with different thicknesses. The dielectric layer was made of aluminum oxide, which was fabricated by atomic layer deposition at thicknesses of 3.8, 7.3, 14.4, and 26 nm (Figure 5a).^26^ The scattering spectra of the L-handed CGNCs supported on the Au/Al_2_O_3_ bilayer substrates were first measured under the excitation of unpolarized light (Figure S16a). The intensity of the scattering peak in the wavelength range of 800–950 nm is dramatically enhanced when the thickness of the dielectric layer is increased from 14.4 to 26.0 nm. The simulated scattering spectra of the CGNCs supported on the bilayer substrates show that the scattering peak of M_3_ appears when the Al_2_O_3_ layer thickness is 14.4 nm and is blue-shifted as the thickness is increased (Figure S16b). The scattering spectra under the excitation of CPL and scattering *g*-factor spectra of the CGNCs with *L* = ∼150 nm supported on the bilayer substrates were measured. Take the D-handed CGNC as an example. As the Al_2_O_3_ layer thickness is increased from 3.8 to 7.3 nm, the *g*-factor dip is blue-shifted from 690 to 648 nm, and their absolute values decrease from 0.61 to 0.31 (Figure 5b). The *g*-factor becomes positive when the thickness of the Al_2_O_3_ layer is 26.0 nm. The *g*-factor spectra of the D- and L-handed CGNCs supported on the bilayer substrates show good mirror symmetry (Figures 5b and S17). The *g*-factor spectra for the D- and L-handed CGNCs on the bilayer substrates were averaged over 20 CGNCs (Figure 5c). The average *g*-factor bands of the Au/Al_2_O_3_-supported CGNCs are blue-shifted, vanished, and inverted as the Al_2_O_3_ layer thickness is increased from 3.8 to 26.0 nm. Therefore, the effect of the Au substrate on the chiroptical response can be modulated by the introduction of a dielectric layer between the Au film and the chiral NPs.

We further employed effective medium theory to explore the chiroptical properties of the chiral NPs on the bilayer substrate. The effective refractive index due to multiple scattering between the two interfaces is
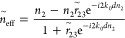
6where , *d*, *n*_2_, and *k*_0_ are the reflection coefficient at the interface between the spacer and the Au film, the thickness of the spacer, the refractive index of the spacer, and the vacuum wavenumber of light, respectively.^27^ The ratio of *g*/*g*_0_ becomes [(1 + *q*) Re ()]/(1 + *q*||^2^) for a bilayer substrate with an effective dielectric function of  = . The chiroptical signals of the chiral NPs on the Au/Al_2_O_3_ substrates therefore vary with the thickness of the Al_2_O_3_ layer. The ratios of *g*/*g*_0_ for the CGNC supported on the Au/Al_2_O_3_ substrates were calculated by the use of FDTD to compare with those evaluated based on the effective medium theory. The simulated scattering *g*-factor spectra of the D-handed CGNCs and the corresponding *g*/*g*_0_ are shown in Figure S18a,b. Figure S18b demonstrates that the dip value of *g*/*g*_0_ for the structure with a 3.8 nm thick Al_2_O_3_ layer reaches −23 at a wavelength of 916 nm. The wavelength of the *g*/*g*_0_ dip blue-shifts, and the value of the *g*/*g*_0_ dip increases in the wavelength range of 769–988 nm as the thickness of the Al_2_O_3_ layer is increased from 3.8 to 26 nm. Similar simulations and calculations were performed for the L-handed CGNCs supported on the Au/Al_2_O_3_ substrates (Figure S18c,d). The *g*/*g*_0_ spectra for the D-handed and L-handed structures demonstrate consistent variations with the thickness of the Al_2_O_3_ layer. The spectra of (*g*/*g*_0_)_theo_ calculated based on the effective medium theory are displayed in Figure 5d–f. The thickness of the Al_2_O_3_ layer is increased from 3.8 to 14.4 nm, which results in decreases in the absolute value of the (*g*/*g*_0_)_theo_ dip, together with blue-shifts in the dip wavelength (Figure 5d). When the thickness of the Al_2_O_3_ layer is 25 nm, a *y*-axis zero-crossing point appears at 710 nm, and the values of (*g*/*g*_0_)_theo_ become positive in the spectral range of 710–886 nm (Figure 5f). As the thickness is increased from 25 to 30 nm, one of the zero-crossing points is blue-shifted from the wavelength of 886 to 598 nm. The values of (*g*/*g*_0_)_theo_ become positive in the wavelength range of 614–752 nm when the thickness is 26 nm. Comparison of the calculated results in Figure S18b,d with the theoretical results in Figure 5d–f reveals that the effective medium theory is valid in the wavelength range under the assumption that the factor *q* can be treated as a constant. Figure S19 shows the spectra of (*g*/*g*_0_)_s_ and (*g*/*g*_0_)_p_ for the Au/Al_2_O_3_ substrates under the excitation of the s- and p-polarized components of CPL at an incidence angle of 64° (see Methods). Such an effective medium theory provides an approach for analyzing the *g*-factors of chiral NPs supported on multilayer substrates, which is also applicable for predicting the chiroptical responses of chiral systems composed of complicated components.

## Conclusion

In summary, we have systematically investigated the chiroptical responses of the CGNCs supported on different substrates, including SiO_2_, Au, and Au/Al_2_O_3_. When a CGNC is transferred from the SiO_2_ substrate to the Au film, the scattering *g*-factor of the CGNC is inverted with enhanced intensity. Such a sign inversion of the chiroptical signal is essential for polarization engineering and information storage. To better understand the effect of the supporting substrate on the chiroptical response, we have theoretically considered the mirror dipole resonance induced by the substrate and proposed that the overall chiroptical response of a chiral NP is dependent on the structural chirality of the chiral object itself and the dielectric function of the supporting substrate. If the dielectric constant of the substrate satisfies Re() < 0, it can lead to the inversion of the chiroptical response, as observed in the case of the CGNCs on gold substrates. Conversely, if the dielectric constant of the substrate satisfies Re() > 0, the sign of the *g*-factor should remain the same as that for the CGNCs on SiO_2_ substrates. The electromagnetic field enhancement of the CGNCs supported on Au films results in enhanced scattering intensity and stronger differential scattering. The calculated optical chirality distributions in a region above the CGNC reveal that the optical chirality is inverted and enhanced when the substrate is changed from SiO_2_ to Au. The calculation results lead to the possibility that the absorption *g*-factor of chiral molecules placed in the region will be inverted and enhanced by the modulation of the dielectric properties of the substrate. When chiral plasmonic nanostructures, composed of chiral molecules and plasmonic components, are deposited on substrates, the chiroptical response is affected by the near-field distribution of the optical chirality, the interaction of the electromagnetic field with the substrate material and the plasmonic nanostructure, and the intrinsic absorption *g*-factor of the chiral molecules. On the other hand, the variation of the *g*-factor band with the effective dielectric function of the bilayer substrate has been further confirmed. Such an effect is applicable to the chiroptical responses of chiral NPs on multilayer substrates.

Our approach to chiroptical inversion offers convenience and universality, which is distinct from other methods that rely on structural changes in NP assemblies.^23^ First, inversion can be realized by various substrates as long as the real part of the dielectric constant is negative. Second, the integration of substrates with existing technologies becomes feasible for diverse applications in sensing, imaging, and optoelectronics. Third, the inversion of the chiroptical response with a gold film significantly amplifies the chiroptical signal, thereby enhancing the sensitivity of the chiroptical measurements. In the immediate future, an exhaustive study is required to create ultrasensitive chiral membranes by utilizing amplified chiroptical signals. For example, combining stretchable substrates with chiral NPs can enable chiral interfaces with continuously modulable chiroptical responses under external stimuli. The nature of chiral nanocavities offers the potential for chiral coupling between light and quantum emitters in nanophotonic structures. Such chiral light–matter interaction extends plasmonic nanocavities to the application scenarios with more degrees of freedom and better adaptivity.

## Methods

### Synthesis of the Chiral Nanoparticles

The CGNCs were synthesized by using a two-step wet-chemistry method. A seed-mediated growth method was used to produce Au nano-octahedra with an edge size of ∼30 nm, followed by centrifugation and redispersion in deionized (DI) water.^28^ The CGNCs were grown from the nano-octahedra in a mixture solution containing CTAB (0.1 M), ascorbic acid (0.1 M), HAuCl_4_ (0.01 M), GSH (2.75 mM), and DI water. After 3 h of storage at 35 °C, the mixture solution turned red. The GSH molecules tethered to the Au surface controlled the chirality of the high-Miller-index facets, resulting in cubic NPs with four highly curved arms on each facet. The sizes of the CGNCs were adjusted by changing the amount of HAuCl_4_ in the overgrowth process. The volumes of the HAuCl_4_ solution were 600, 800, and 1000 μL for the synthesis of the CGNCs with *L* values of 150, 170, and 190 nm, respectively.

### Electrodynamic Simulations

The electromagnetic simulations were performed using FDTD Solutions 2020 R2 (Lumerical). During the simulations, a total-field scattered-field (TFST) source was launched into a box containing a CGNC placed on a substrate. An etched cube model was used to emulate the morphology of the actual CGNC as closely as possible. A mesh size of 2 nm was employed in the simulations. The substrate was modeled as a thin cuboid. As the residual CTAB molecules did not get completely removed from the nanoparticles in our experiments, the CTAB layer with a thickness of 1 nm between the CGNC and the substrate was taken into account in the simulations.^29^ The refractive index and thickness of the SiO_2_ substrate were set at 1.45 and 300 nm, respectively. The thickness of the Au film was 100 nm. The dielectric function of Au was taken from Johnson and Christy’s data.^35^ The bilayer substrates for the calculations of the supported CGNCs were composed of Al_2_O_3_, Au, and CTAB. The dielectric function of Al_2_O_3_ was calculated by fitting the experimental data of Palik.^36^ CPL was produced when the two orthogonal electric field component vectors were of equal magnitude and out of phase by 90°. The phase of the *x*-polarized plane wave was fixed at 0°. A positive phase difference of +90° between the *y*- and *x*-polarized plane waves gave LCP light. A negative phase difference of −90° gave RCP light. The region of interest for the simulations of the scattering power and the distributions of the optical chirality was located 130 nm above the top surface of the CGNC and outside the box of the TFST source. The optical chirality is expressed as *C* = −ε_0_ω Im[ · ]/2,^30^ with , , ε_0_, and ω being the electric field, magnetic field, permittivity of free space, and angular frequency of light, respectively.

### Single-Particle Optical Measurements and Characterization

An optical microscope (Olympus, BX53M) equipped with a monochromator (Acton, SpectraPro 2360i) and a charge-coupled device camera (Princeton Instruments, Pixis 400, cooled to −70 °C) was used to measure the dark-field scattering spectra. A 100× dark-field air objective (Olympus, numerical aperture: 0.9) was used for scattering measurements. Light from a halogen lamp passed through the objective and illuminated the sample obliquely. The backward scattered light passed through the same objective. Dark-field differential scatterometry can provide richer information than optical measurements of the solution samples containing the randomly orientated CGNCs. Circularly polarized excitation in our experiments was realized by a linear polarizer and a quarter-wave plate, both of which were purchased from Union Optic. The working wavelength of the quarter-wave plate (WPA4420–550–750) is 550–750 nm. The polarization handedness convention used in this work is such that the RCP and LCP vectors rotate clockwise and counterclockwise along the propagation axis, respectively. The wavelengths of the scattering peaks were extracted by fitting the scattering spectra with Gaussian functions. Extinction spectra were measured on a PerkinElmer Lambda 950 ultraviolet/visible/near-infrared spectrophotometer. SEM imaging was carried out on an FEI QF400 field-emission scanning electron microscope operated at a rate of 20 kV.

### Transferring of the Individual Chiral Nanoparticles

Electron-beam evaporation (EBS-500, Junsun Tech Co., Taiwan) was used to deposit 100 nm thick Au films onto smooth Si substrates. The ultraflat Au films were fabricated through a template-exfoliation process.^31^ During precleaning, Si wafers were submerged in an acetone bath under ultrasonication for 10 min, followed by 5 min of ultrasonication treatment in isopropanol. The Si wafer was then blown dry with nitrogen and baked on a hot plate at 120 °C for 5 min. A two-component thermal epoxy (EPO-TEK 377) was subsequently spin-coated over a precleaned 8 × 8 mm glass slide and baked for 1 h at 150 °C. The Au film was then peeled off slowly with the glass slide glued to it. As a result, the ultraflat Au surface, which was originally in contact with the Si substrate, was exposed to further use. The CGNCs were rinsed twice and diluted with DI water at a particle concentration of ∼1 pM. The CGNC solution (5 μL) was dropped onto a Si substrate capped with a 300 nm thick SiO_2_ layer. The substrate was then dried with nitrogen. The CGNCs deposited on the Si/SiO_2_ substrate were transferred onto the ultraflat Au film after close contact between the surfaces of the Si/SiO_2_ substrate and the Au film at 90 °C for 20 min.

### Contribution of the Electric Quadrupole Mode to the Chiroptical Response

The generated dipolar and higher-order moments by a CGNC on a substrate can be described by an effective ED moment , MD moment , and EQ moment , with contributions from both the chiral NP and the image chiral NP (Figure S20).^32,33^ The components of the EQ moment of an NP on a substrate are  = (1 – *K*),  = (1 – *K*), and  = (1 + *K*). The superscripts *yz*, *xz*, and *xy* represent the planes of the charges distributed with respect to the EQ moment components. The contributions from the ED and EQ moments to the CD response are of the form^34^
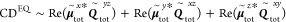
7

The plane of the substrate is defined as the *xy*-plane.  and  can be defined to be .  can be defined to be . || and ⊥ refer to the normal directions of the planes of the charges oriented parallel and perpendicular to the substrate surface, respectively. The total ED and EQ moments of the chiral NP supported on a substrate can therefore be written as eq 1 and
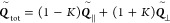
8

The *g*-factor of a chiral NP supported on a substrate can therefore be calculated according to
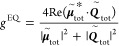
9*g*_0_ is the *g*-factor of the chiral NP in vacuum. It can be expressed as
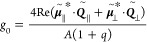
10where *q* = *B*/*A*, with *A* = ||^2^ + ||^2^ and *B* = ||^2^ + ||^2^. The equation of *g*^EQ^/*g*_0_ therefore has the form
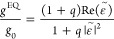
11

It is important to consider higher-order modes, such as the EQ mode, especially in the near-field where the electric field gradient near the NP can be significant. However, for far-field optical responses, the contribution from the EQ moment to the chiroptical response averages out over all orientations.^34^ In our single-particle circular differential scattering measurements, we employed oblique incident light by focusing a ring-shaped beam onto the chiral NP (Figure S3). This configuration partially cancels out the contribution from the EQ moment, since the incident CPL arrives from multiple symmetric directions. To validate this hypothesis, we performed additional calculations on the multipolar expansion of the plasmon modes in an L-handed CGNC under the excitation of two opposite linearly polarized light beams (Figure S21).

### Calculation of the Effective Refractive Index

The Fresnel equations for the s- and p-components of the reflection coefficients can be used for obliquely incident CPL. At any instant of time, the electric field vector of the CPL rotates at an angular velocity in a plane perpendicular to the **k** vector. CPL can be decomposed into two equal s- and p-polarized components with orthogonal polarization directions and a relative phase shift of π/2. The effective refractive index  due to multiple scattering for the s-polarized component of obliquely incident CPL is
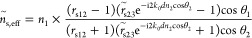
12

The effective refractive index  due to multiple scattering for the p-polarized component of obliquely incident CPL is
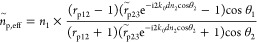
13

In the above two equations, *r*_12_ is the reflection coefficient of the spacer in air,  is the reflection coefficient at the interface between the spacer and the Au film, θ_1_ is the incidence angle of light propagating from air to the spacer, θ_2_ is the refraction angle of light propagating from the spacer to the Au film, *d* is the thickness of the spacer, and *n*_1_, *n*_2_, and *k*_0_ are the refractive index of air, the refractive index of the spacer, and the vacuum wavenumber of light, respectively. The footnotes s and p represent that the values are calculated under the excitation of the s- and p-polarized components of CPL, respectively. Next, we can calculate the ratio of (*g*/*g*_0_)_s_ and (*g*/*g*_0_)_p_ for the s- and p-polarized components of obliquely incident CPL according to
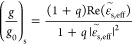
14
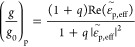
15 and  are the effective dielectric constants of the multiplayer substrate for the s- and p-polarized components of obliquely incident CPL, respectively. They can be expressed as  =  and  = .

## Supporting Information Available

Morphologies of the chiral nanoparticles; simulation of a CGNC immersed in water; dark-field scattering measurements; inversion of the scattering *g*-factor spectra; simulations of the CGNC supported on substrates; simulated scattering cross-section spectra of the CGNCs; SEM images, scattering, and *g*-factor spectra of the CGNCs with different sizes; scattering and *g*-factor spectra of the CGNCs supported on the Au/Al_2_O_3_ substrates with different Al_2_O_3_ layer thicknesses; effect of the dielectric function of the substrate on the chiroptical response; multipolar expansion of the plasmon modes in the CGNCs (PDF)

## References

[ref1] Ma W.; Xu L. G.; de Moura A. F.; Wu X. L.; Kuang H.; Xu C. L.; Kotov N. A. Chiral inorganic nanostructures. Chem. Rev. 2017, 117, 8041–8093. 10.1021/acs.chemrev.6b00755. 28426196

[ref2] Hentschel M.; Schäferling M.; Duan X. Y.; Giessen H.; Liu N. Chiral Plasmonics. Sci. Adv. 2017, 3, e1602735 10.1126/sciadv.1602735. 28560336 PMC5435411

[ref3] Lee H. E.; Ahn H. Y.; Mun J.; Lee Y. Y.; Kim M.; Cho N. H.; Chang K.; Kim W. S.; Rho J.; Nam K. T. Amino-Acid- and Peptide-Directed Synthesis of Chiral Plasmonic Gold Nanoparticles. Nature 2018, 556, 360–365. 10.1038/s41586-018-0034-1. 29670265

[ref4] Wen Y.; He M. Q.; Yu Y. L.; Wang J. H. Biomolecule-Mediated Chiral Nanostructures: A Review of Chiral Mechanism and Application. Adv. Colloid Interface Sci. 2021, 289, 102376 10.1016/j.cis.2021.102376. 33561566

[ref5] Zheng G. C.; He J. J.; Kumar V.; Wang S. L.; Pastoriza-Santos I.; Pérez-Juste J.; Liz-Marzán L. M.; Wong K. Y. Discrete metal nanoparticles with plasmonic chirality. Chem. Soc. Rev. 2021, 50, 3738–3754. 10.1039/C9CS00765B. 33586721

[ref6] Wu W.; Pauly M. Chiral plasmonic nanostructures: recent advances in their synthesis and applications. Mater. Adv. 2022, 3, 186–215. 10.1039/D1MA00915J.

[ref7] Wang S. L.; Liu X.; Mourdikoudis S.; Chen J.; Fu W. W.; Sofer Z.; Zhang Y.; Zhang S. P.; Zheng G. C. Chiral Au nanorods: synthesis, chirality origin, and applications. ACS Nano 2022, 16, 19789–19809. 10.1021/acsnano.2c08145. 36454684

[ref8] Collins J. T.; Kuppe C.; Hooper D. C.; Sibilia C.; Centini M.; Valev V. K. Chirality and chiroptical effects in metal nanostructures: fundamentals and current trends. Adv. Opt. Mater. 2017, 5, 1700182 10.1002/adom.201700182.

[ref9] Banik M.; Rodriguez K.; Hulkko E.; Apkarian V. A. Orientation-dependent handedness of chiral plasmons on nanosphere dimers: How to turn a right hand into a left hand. ACS Photonics 2016, 3, 2482–2489. 10.1021/acsphotonics.6b00733.

[ref10] Gansel J. K.; Thiel M.; Rill M. S.; Decker M.; Bade K.; Saile V.; von Freymann G.; Linden S.; Wegener M. Gold helix photonic metamaterial as broadband circular polarizer. Science 2009, 325, 1513–1515. 10.1126/science.1177031. 19696310

[ref11] Chen Y.; Gao J.; Yang X. D. Direction-controlled bifunctional metasurface polarizers. Laser Photonics Rev. 2018, 12, 1800198 10.1002/lpor.201800198.

[ref12] Banzer P.; Woźniak P.; Mick U.; De Leon I.; Boyd R. W. Chiral optical response of planar and symmetric nanotrimers enabled by heteromaterial selection. Nat. Commun. 2016, 7, 13117 10.1038/ncomms13117. 27734960 PMC5065623

[ref13] Wagenknecht C.; Li C. M.; Reingruber A.; Bao X. H.; Goebel A.; Chen Y. A.; Zhang Q.; Chen K.; Pan J. W. Experimental demonstration of a heralded entanglement source. Nat. Photonics 2010, 4, 549–552. 10.1038/nphoton.2010.123.

[ref14] Kim Y.; Kim H.; Yang Y.; Badloe T.; Jeon N.; Rho J. Three-dimensional artificial chirality towards low-cost and ultra-sensitive enantioselective sensing. Nanoscale 2022, 14, 3720–3730. 10.1039/D1NR05805C. 35230363

[ref15] Spreyer F.; Mun J.; Kim H.; Kim R. M.; Nam K. T.; Rho J.; Zentgraf T. Second harmonic optical circular dichroism of plasmonic chiral helicoid-III nanoparticles. ACS Photonics 2022, 9, 784–792. 10.1021/acsphotonics.1c00882. 35330905 PMC8932316

[ref16] Duan T. W.; Ai J.; Chen S. J.; He G. F.; Guo X. J.; Han L.; Che S. A.; Duan Y. Y. Chiral CdSe/CdS quantum dot (in rod)-light-emitting diodes with circularly polarized electroluminescence. Nano Res. 2022, 15, 9573–9577. 10.1007/s12274-022-4536-7.

[ref17] Karimullah A. S.; Jack C.; Tullius R.; Rotello V. M.; Cooke G.; Gadegaard N.; Barron L. D.; Kadodwala M. Disposable plasmonics: plastic templated plasmonic metamaterials with tunable chirality. Adv. Mater. 2015, 27, 5610–5616. 10.1002/adma.201501816. 26306427

[ref18] Lee S.; Lim Y. C.; Kim H.; Seo D. H.; Na J.; Kim H.; Nam K. T.; Jeong Y. Random lasing with a high degree of circular dichroism by chiral plasmonic gold nanoparticles. ACS Photonics 2022, 9, 613–620. 10.1021/acsphotonics.1c01601.

[ref19] Nechayev S.; Barczyk R.; Mick U.; Banzer P. Substrate-induced chirality in an individual nanostructure. ACS Photonics 2019, 6, 1876–1881. 10.1021/acsphotonics.9b00748.

[ref20] Chikkaraddy R.; De Nijs B.; Benz F.; Barrow S. J.; Scherman O. A.; Rosta E.; Demetriadou A.; Fox P.; Hess O.; Baumberg J. J. Single-molecule strong coupling at room temperature in plasmonic nanocavities. Nature 2016, 535, 127–130. 10.1038/nature17974. 27296227 PMC4947385

[ref21] Baumberg J. J.; Aizpurua J.; Mikkelsen M. H.; Smith D. R. Extreme nanophotonics from ultrathin metallic gaps. Nat. Mater. 2019, 18, 668–678. 10.1038/s41563-019-0290-y. 30936482

[ref22] Tang Y. Q.; Cohen A. E. Optical chirality and its interaction with matter. Phys. Rev. Lett. 2010, 104, 163901 10.1103/PhysRevLett.104.163901. 20482049

[ref23] Mun J.; Kim M.; Yang Y.; Badloe T.; Ni J. C.; Chen Y.; Qiu C. W.; Rho J. Electromagnetic chirality: from fundamentals to nontraditional chiroptical phenomena. Light: Sci. Appl. 2020, 9, 139 10.1038/s41377-020-00367-8. 32922765 PMC7463035

[ref24] Hu L.; Tian X. R.; Huang Y. Z.; Fang L.; Fang Y. R. Quantitatively analyzing the mechanism of giant circular dichroism in extrinsic plasmonic chiral nanostructures by tracking the interplay of electric and magnetic dipoles. Nanoscale 2016, 8, 3720–3728. 10.1039/C5NR08527F. 26814829

[ref25] Noginova N.; Hussain R.; Noginov M. A.; Vella J.; Urbas A. Modification of electric and magnetic dipole emission in anisotropic plasmonic systems. Opt. Express 2013, 21, 23087–23096. 10.1364/OE.21.023087. 24104224

[ref26] Cui X. M.; Lai Y. H.; Ai R. Q.; Wang H.; Shao L.; Chen H. J.; Zhang W.; Wang J. F. Anapole states and toroidal resonances realized in simple gold nanoplate-on-mirror structures. Adv. Opt. Mater. 2020, 8, 2001173 10.1002/adom.202001173.

[ref27] Harbecke B. Coherent and incoherent reflection and transmission of multilayer structures. Appl. Phys. B: Photophys. Laser Chem. 1986, 39, 165–170. 10.1007/BF00697414.

[ref28] Wu H. L.; Tsai H. R.; Hung Y. T.; Lao K. U.; Liao C. W.; Chung P. J.; Huang J. S.; Chen I. C.; Huang M. H. A comparative study of gold nanocubes, octahedra, and rhombic dodecahedra as highly sensitive SERS substrates. Inorg. Chem. 2011, 50, 8106–8111. 10.1021/ic200504n. 21797229

[ref29] He J.; Unser S.; Bruzas I.; Cary R.; Shi Z. W.; Mehra R.; Aron K.; Sagle L. The facile removal of CTAB from the surface of gold nanorods. Colloids Surf., B 2018, 163, 140–145. 10.1016/j.colsurfb.2017.12.019. 29291499

[ref35] Johnson P. B.; Christy R. W. Optical constants of the noble metals. Phys. Rev. B 1972, 6, 4370–4379.

[ref36] Handbook of optical constants of solids Palik E. D., ed. Academic Press, 1998.

[ref30] Schäferling M.; Dregely D.; Hentschel M.; Giessen H. Tailoring enhanced optical chirality: design principles for chiral plasmonic nanostructures. Phys. Rev. X 2012, 2, 031010 10.1103/PhysRevX.2.031010.

[ref31] Rueda A.; Vogel N.; Kreiter M. Characterization of gold films by surface plasmon spectroscopy: large errors and small consequences. Surf. Sci. 2009, 603, 491–497. 10.1016/j.susc.2008.12.006.

[ref32] Wu T.; Zhang W. X.; Wang R. Y.; Zhang X. D. A giant chiroptical effect caused by the electric quadrupole. Nanoscale 2017, 9, 5110–5118. 10.1039/C6NR09419H. 28387409

[ref33] Hinamoto T.; Fujii M. MENP: an open-source MATLAB implementation of multipole expansion for nanophotonics. Osa Continuum 2021, 4, 1640–1648. 10.1364/OSAC.425189.

[ref34] Buckingham A. D.; Dunn M. B. Optical activity of oriented molecules. J. Chem. Soc. A 1971, 1988–1991. 10.1039/j19710001988.

