# Supplementary material for: Inversion of the Chiroptical Responses of Chiral Gold Nanoparticles with a Gold Film
Source: ACS Nano. 2023 Dec 21;18(1):383–94. doi: 10.1021/acsnano.3c07475 (PMC10786168; doi:10.1021/acsnano.3c07475)
Supplement: Supplementary file 1 — nn3c07475_si_001.pdf [file nn3c07475_si_001.pdf]

Supporting Information

Inversion of the Chiroptical Responses of Chiral Gold Nanoparticles with a Gold Film

Yilin Chen,[1] *Jiapeng Zheng,*[1] *Lingling Zhang,*[1] *Shasha Li,*[1] *Yang Chen,*[1] *Ka Kit Chui,*[1] *Wei Zhang,*[2,]* *Lei Shao,*[3] *and Jianfang Wang*[1,]*

[1]Department of Physics, The Chinese University of Hong Kong, Shatin, Hong Kong SAR 999077, China

[2]Institute of Applied Physics and Computational Mathematics, Beijing 100088, China

[3]State Key Laboratory of Optoelectronic Materials and Technologies, Guangdong Province Key Laboratory of Display Material and Technology, School of Electronics and Information Technology, Sun Yat-sen University, Guangzhou 510275, China

*Email: zhang_wei@iapcm.ac.cn; jfwang@phy.cuhk.edu.hk

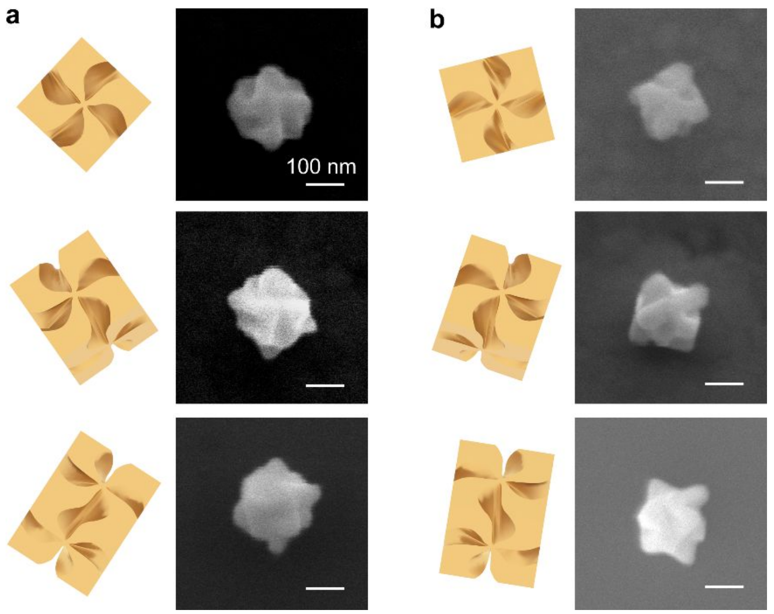

Figure S1. Morphologies of the chiral nanoparticles. (a,b) Scanning electron microscopy (SEM) images of the D- (a) and L-handed (b) chiral gold nanocubes (CGNCs) deposited on Si/SiO_2 substrates. As we trace a half-ring of the arm from the core to the end, the direction of the curvature of the twisty arm can be assigned as clockwise or counterclockwise, corresponding to the D- and L-handed CGNCs. The third SEM images in (a) and (b) show the distorted rhombus boundaries along the {100} direction of the CGNCs. Scale bars, 100 nm.

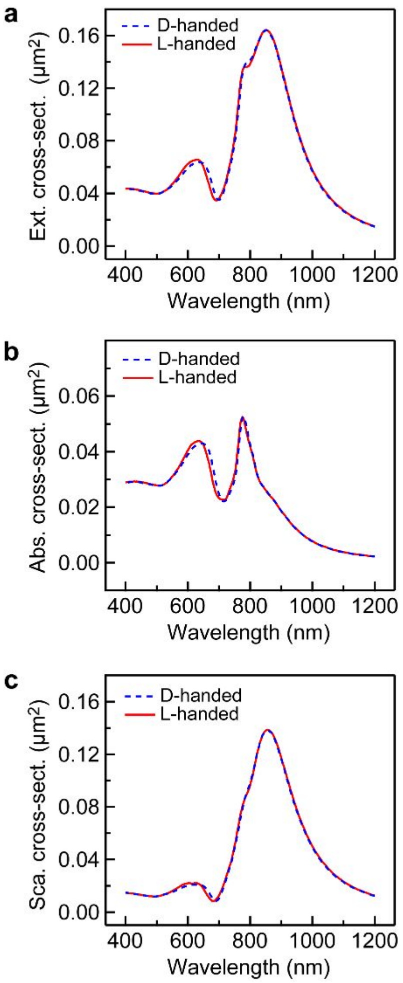

Figure S2. Simulation of a CGNC immersed in water. (a–c) Simulated extinction (a), absorption (b), and scattering cross-section spectra (c). The dashed and solid lines represent the cross-section spectra of the D- and L-handed CGNCs immersed in a homogeneous medium (n = 1.33), respectively. The edge length (L) of the CGNCs is 130 nm. The L of the CGNC for the simulation is slightly smaller than that of the actual one to allow the calculated results to be consistent with the measured results. The morphological differences are mainly caused by that the corners of the CGNC model are not trimmed. The extinction cross-section spectra of the D- and L-handed CGNCs show three plasmonic resonance peaks in the wavelength range of 400−1200 nm. The two peaks of the scattering cross-sections at 780 nm and 855 nm show higher intensities than those of the absorption cross-sections.

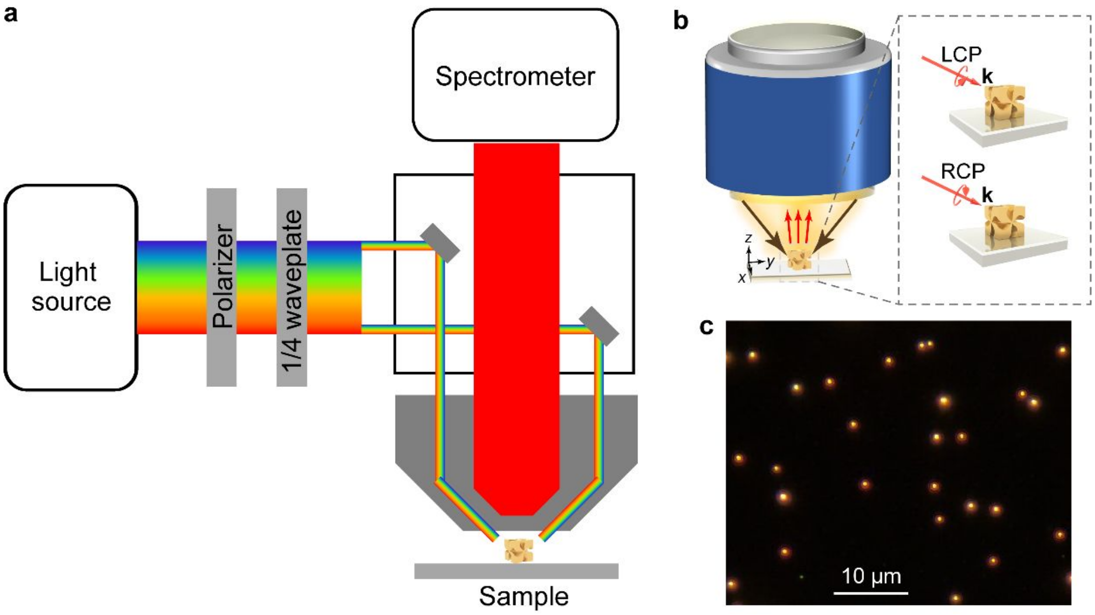

Figure S3. Dark-field scattering measurements. (a,b) Schematics showing the dark-field scattering measurements under the excitation of CPL. (c) Dark-field scattering image of the CGNCs deposited on a Si/SiO_2 substrate.

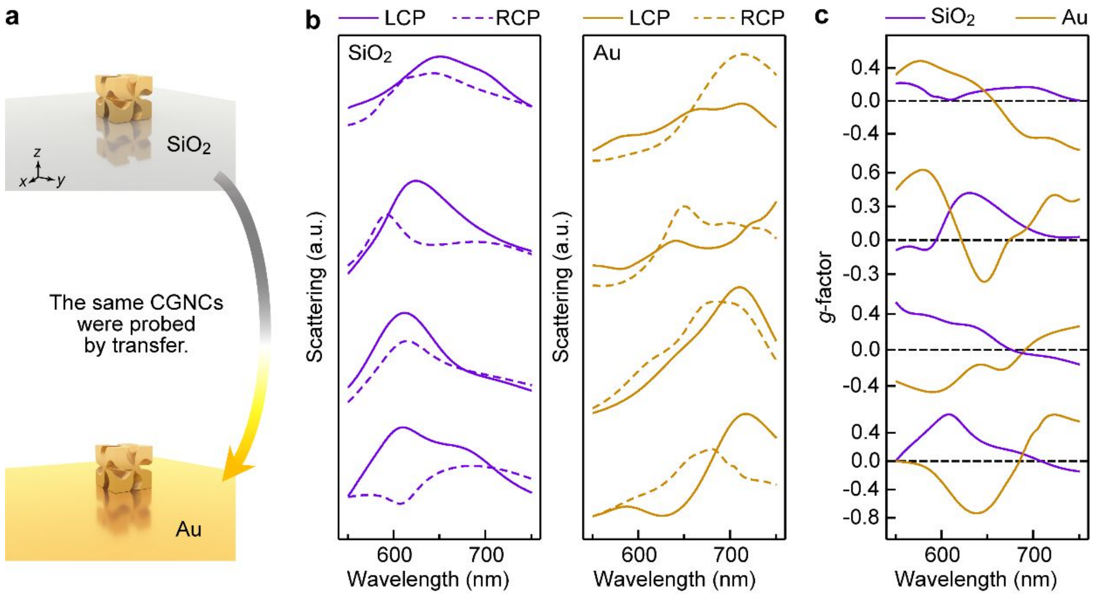

Figure S4. Inversion of the scattering *g*-factor spectra of the D-handed CGNCs. (a) Schematic illustrating the transfer of a single D-handed CGNC from a SiO_2 substrate to a gold film. (b) Scattering spectra of the D-handed CGNCs supported on SiO_2 substrates (purple lines) and Au films (golden lines) under the excitation of CPL. The solid and dashed lines represent the scattering spectra under the excitation of LCP and RCP light, respectively. (c) Scattering *g*-factor spectra of the D-handed CGNCs supported on SiO_2 and Au substrates. The horizontal dashed black lines represent the zero lines of the *g*-factor.

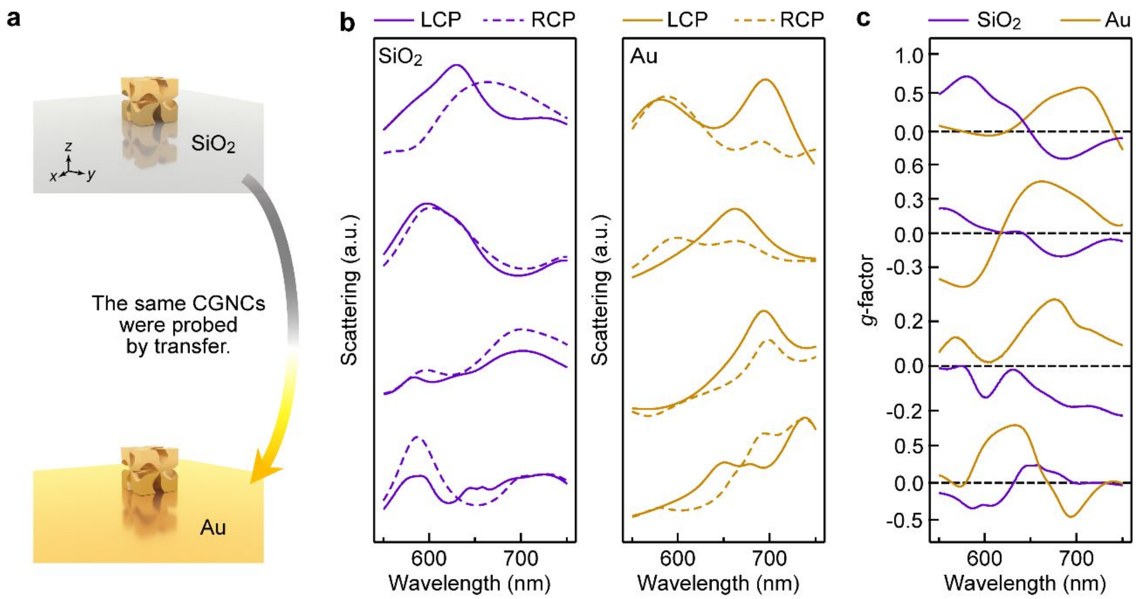

Figure S5. Inversion of the scattering *g*-factor spectra of the L-handed CGNCs. (a) Schematic illustrating the transfer of a single L-handed CGNC. (b) Scattering spectra of the L-handed CGNCs supported on SiO_2 substrates (purple lines) and Au films (golden lines). The solid and dashed lines represent the scattering spectra under the excitation of LCP and RCP light, respectively. (c) Scattering *g*-factor spectra of the L-handed CGNCs supported on SiO_2 and Au substrates.

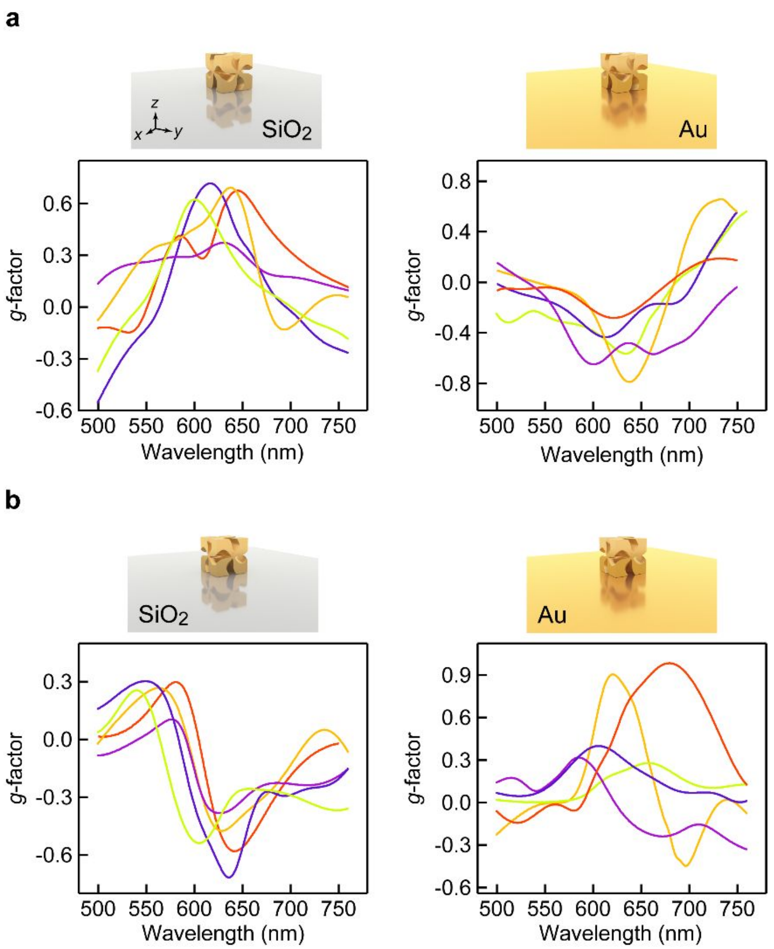

Figure S6. Scattering *g*-factor spectra. (a) Scattering *g*-factor spectra of the D-handed CGNCs supported on SiO_2 and Au substrates. (b) Scattering *g*-factor spectra of the L-handed CGNCs supported on SiO_2 and Au substrates.

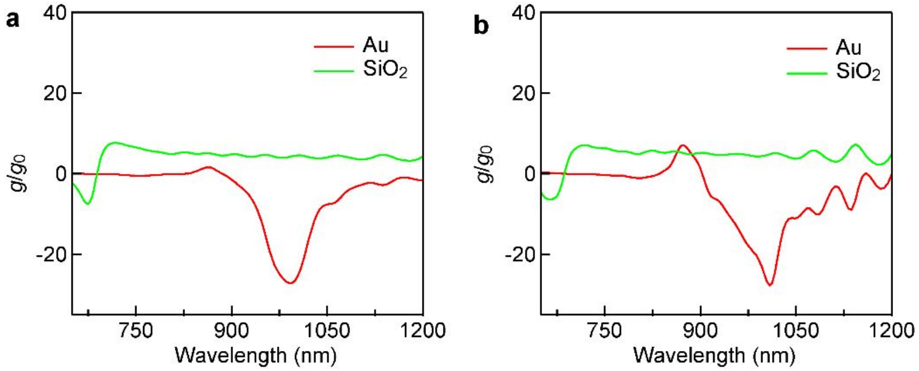

Figure S7. FDTD-simulated g/g_0 for CGNCs supported on the different substrates under normal incidence of CPL. (a) Wavelength-dependent g/g_0 for the results of the D-handed CGNC. (b) Wavelength-dependent g/g_0 for the results of the L-handed CGNC. The green and red lines represent the wavelength-dependent ratios of g_{silica}/g_0 and g_{gold}/g_0, respectively.

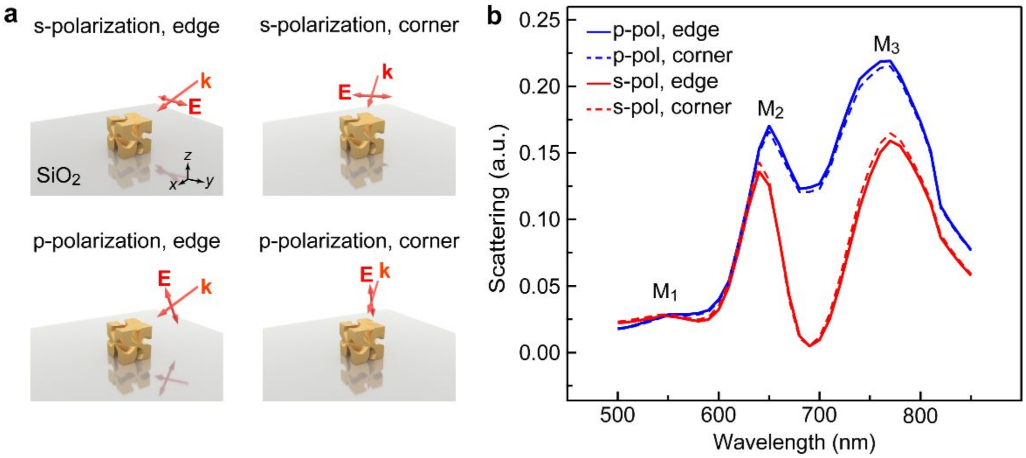

Figure S8. Simulation of the CGNC supported on SiO_2 substrates. (a) Schematics of the CGNC under s- and p-polarized excitation with the excitation light incident towards the edge and corner, respectively. (b) Scattering spectra of the CGNC under the different excitation configurations. To eliminate the effect of the direction of light illuminating at the CGNC on the simulation results, the scattering spectra of the CGNC were simulated under the excitation of light with two incident azimuth angles φ, respectively. One is to let the incidence direction of the plane wave be perpendicular to one edge of the CGNC (i.e., $\varphi = 0°$). The other is to let the incident plane wave point at the corner of the CGNC (i.e., $\varphi = 45°$). For the two situations, the scattering spectra of the CGNC are nearly identical.

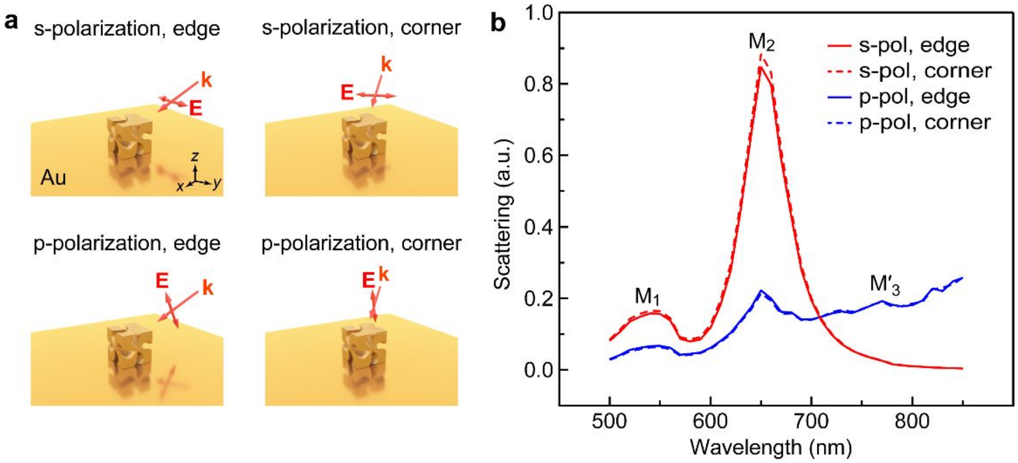

Figure S9. Simulation of the CGNC supported on Au substrates. (a) Schematics of the CGNC under s- and p-polarized excitation with the excitation light incident towards the edge and corner, respectively. (b) Simulated scattering spectra. The in-plane resonance mode contributes to the scattering peak at 661 nm, while the peak appearing in the wavelength range of 700–850 nm originates from the out-of-plane resonance. The simulations of the plane wave illuminating at the edge (i.e., $\varphi = 0°$) and at the corner (i.e., $\varphi = 45°$) of the CGNC supported on Au substrates show similar scattering spectra.

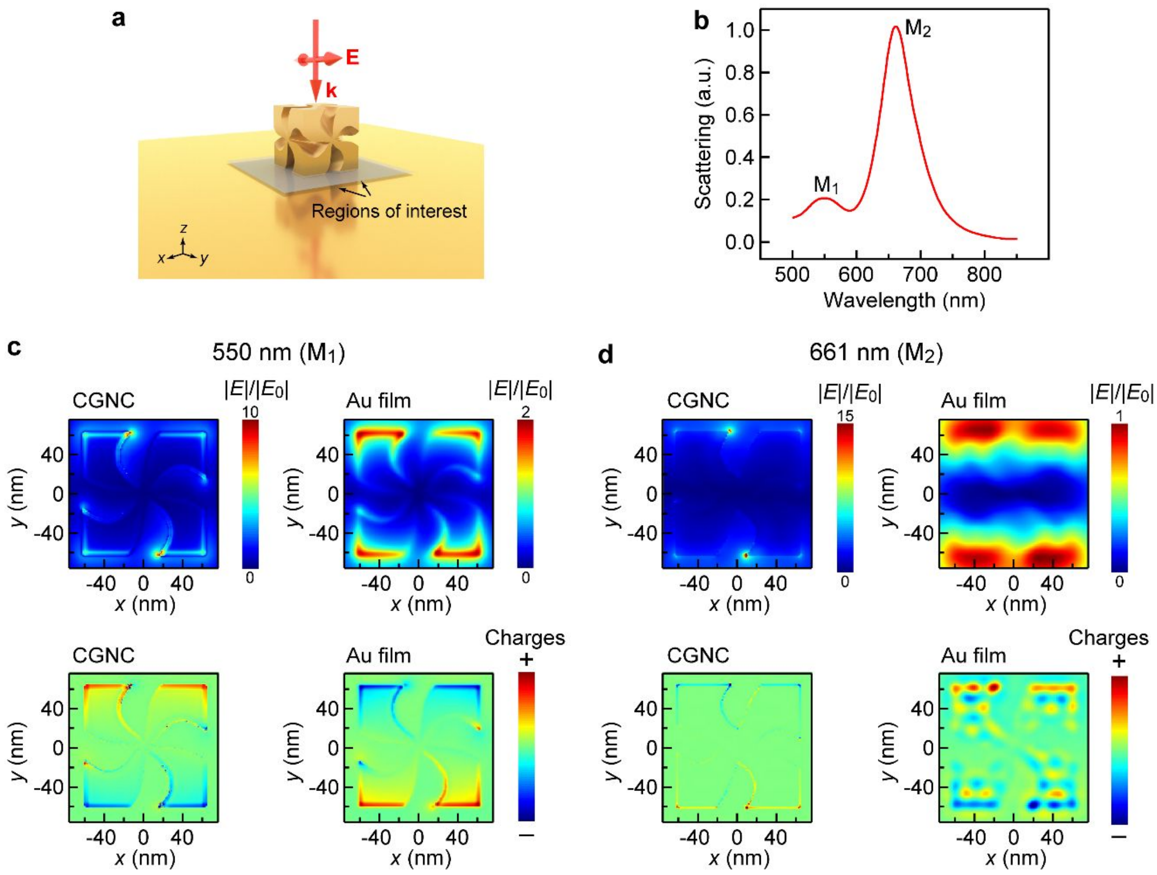

Figure S10. Simulation of the electric field distributions of CGNC-on-Au. (a) Schematic of a CGNC supported on a gold substrate. (b) Simulated scattering spectrum under normal incidence. Two in-plane resonance modes appear at 550 nm (M_1) and 661 nm (M_2). (c,d) Contours of the electric field enhancement and charges in the planes crossing the surface of the CGNC and the surface of the Au film at the wavelengths of 550 nm (c) and 661 nm (d). The charge distributions in the CGNC and the Au film show mirror symmetry with opposite signs. In the Au film, the electric field at the wavelength of 661 nm shows weaker enhancement than that at the wavelength of 550 nm.

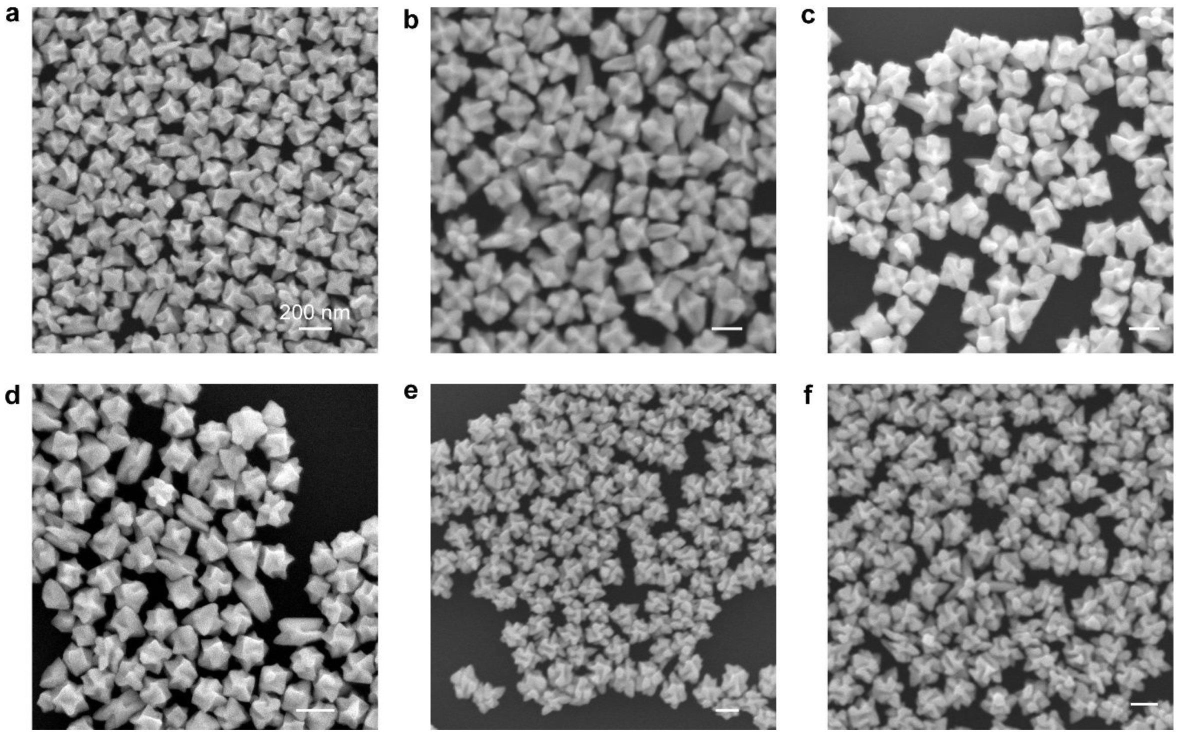

Figure S11. SEM images of the CGNCs with different sizes. (a–c) SEM images of the L-handed CGNSs with average *L* values of ~150, ~170, and ~190 nm, respectively. (d–f) SEM images of the D-handed CGNSs with average *L* values of ~150, ~170, and ~190 nm, respectively. Scale bars, 200 nm.

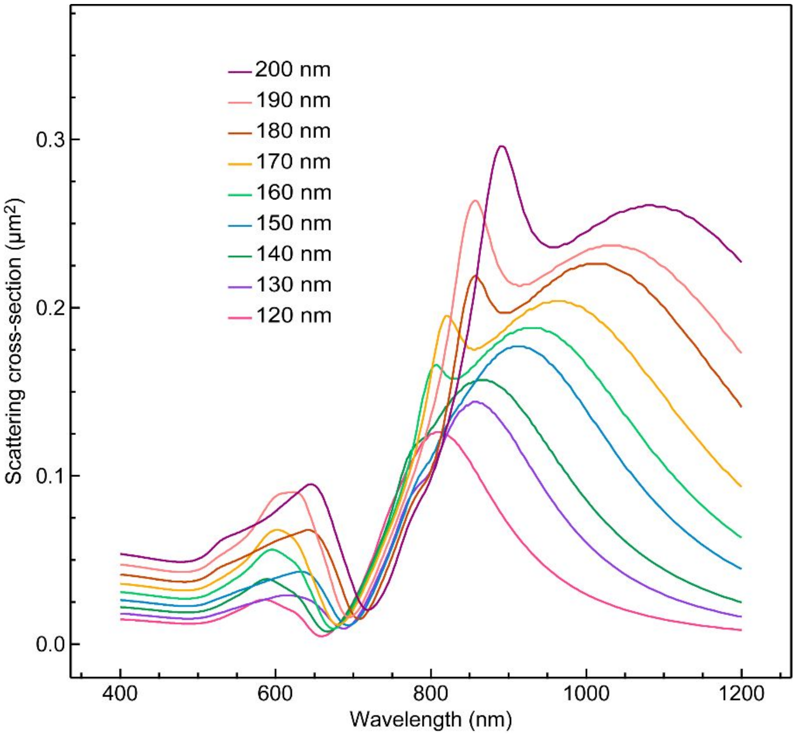

Figure S12. Simulated scattering cross-section spectra of the CGNCs. The CGNCs are immersed in water. The plasmon resonance modes M_2 and M_3 are redshifted with the increase of the L value of the CGNC.

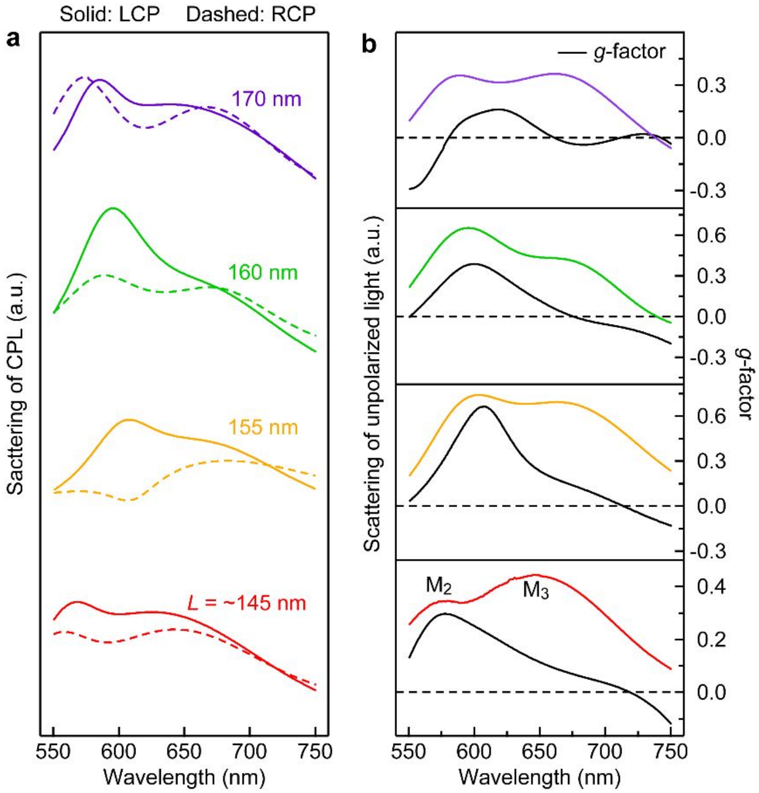

Figure S13. Scattering and *g*-factor spectra of the D-handed CGNCs supported by SiO_2 substrates. (a) Scattering spectra of the CGNCs with different *L* values. The solid and dashed lines represent the scattering spectra under the excitation of LCP and RCP light, respectively. (b) Scattering and *g*-factor spectra. The solid black lines represent the scattering *g*-factor spectra. The horizontal dashed black lines represent the zero lines of the *g*-factor. The red, yellow, green, and purple lines represent the scattering spectra of the CGNCs with different *L* values under the excitation of unpolarized light.

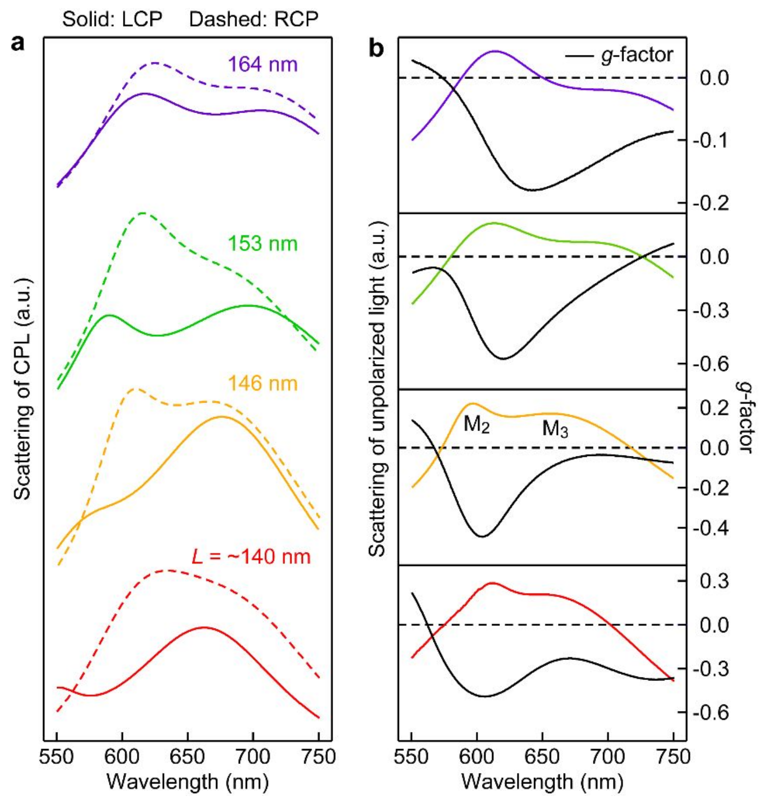

Figure S14. Scattering and *g*-factor spectra of the L-handed CGNCs supported by SiO_2 substrates. (a) Scattering spectra of the CGNCs with different *L* values. The solid and dashed lines represent the scattering spectra under the excitation of LCP and RCP light, respectively. (b) Scattering and *g*-factor spectra. The solid black lines represent the scattering *g*-factor spectra. The horizontal dashed black lines represent the zero lines of the *g*-factor. The red, yellow, green, and purple lines represent the scattering spectra of the CGNCs with different *L* values under the excitation of unpolarized light.

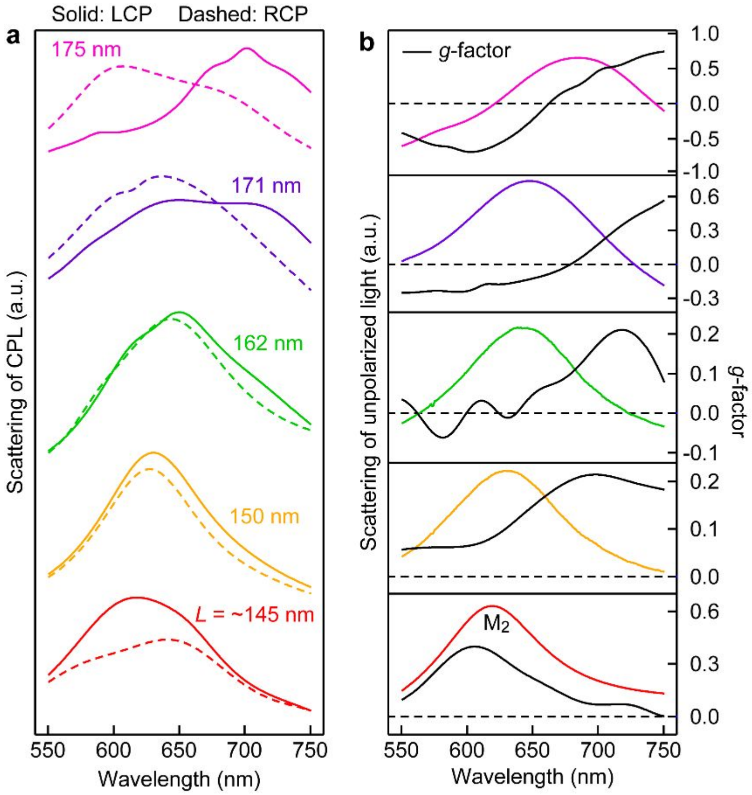

Figure S15. Scattering and *g*-factor spectra of the L-handed CGNCs supported by Au substrates. (a) Scattering spectra of the CGNCs with different *L* values. The solid and dashed lines represent the scattering spectra under the excitation of LCP and RCP light, respectively. (b) Scattering and *g*-factor spectra. The solid black lines represent the scattering *g*-factor spectra. The red, yellow, green, purple, and pink lines represent the scattering spectra of the CGNCs with different *L* values under the excitation of unpolarized light. As the scattering peak is redshifted from 619 nm to 640 nm with the increase of *L* from 145 nm to 162 nm, the *g*-factor peak redshifts from 605 nm to 718 nm. As the scattering peak is redshifted from 648 nm to 684 nm when *L* is increased from 171 nm to 175 nm, the minimum of the *g*-factor decreases from −0.25 to −0.69.

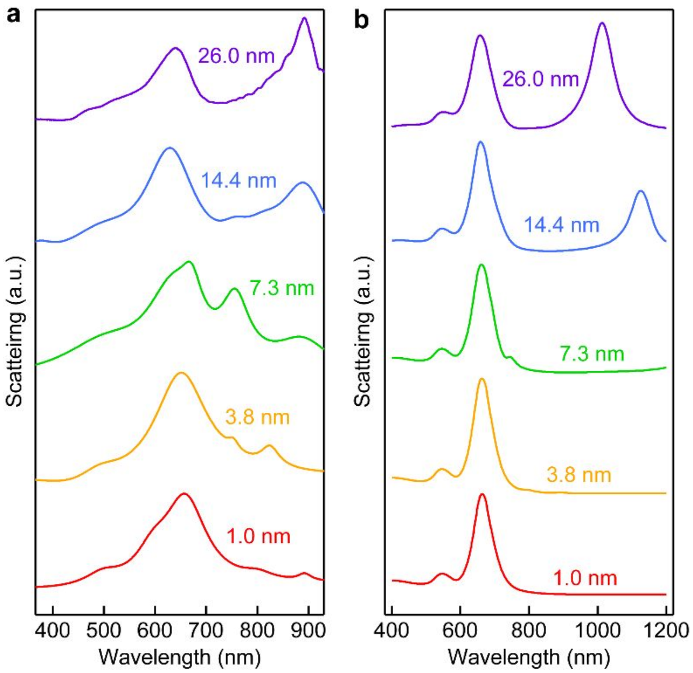

Figure S16. Scattering spectra of the L-handed CGNCs supported on the Au/Al_2O_3 bilayer substrates with Al_2O_3 layer thicknesses of 1.0, 3.8, 7.3, 14.4, and 26.0 nm. (a) Measured scattering spectra under the excitation of unpolarized light. (b) Simulated scattering spectra under the normal incidence of linearly polarized light.

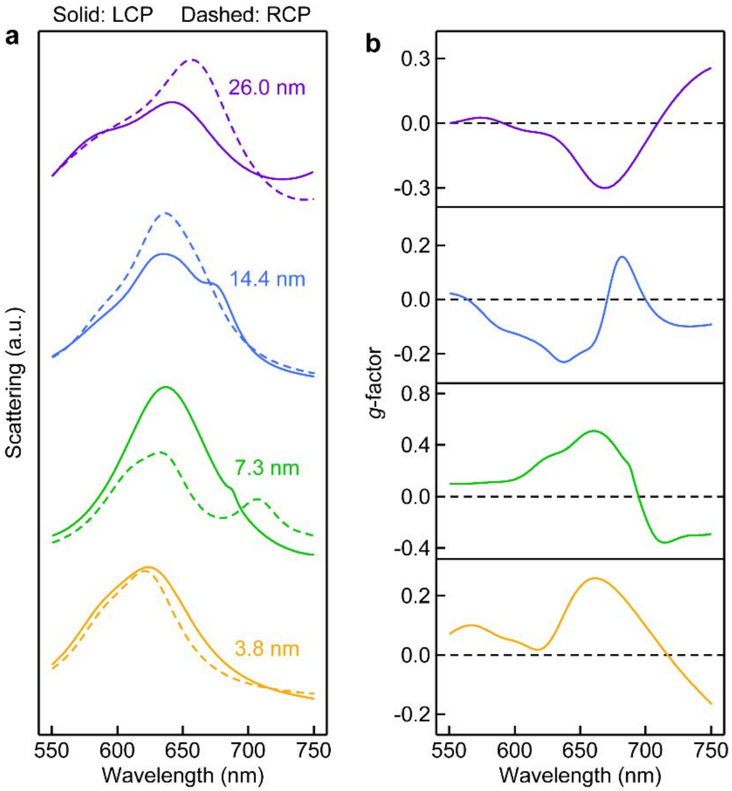

Figure S17. Scattering and *g*-factor spectra of the L-handed CGNCs supported on the Au/Al_2O_3 bilayer substrates with Al_2O_3 layer thicknesses of 3.8, 7.3, 14.4, and 26.0 nm. (a) Scattering spectra. The solid and dashed lines represent the scattering spectra under the excitation of LCP and RCP light, respectively. (b) Scattering *g*-factor spectra. The dashed black lines represent the zero lines of the *g*-factor. The yellow, green, blue, and purple lines represent the *g*-factor spectra of the CGNCs supported on the Au/Al_2O_3 substrates with the corresponding Al_2O_3 layer thicknesses.

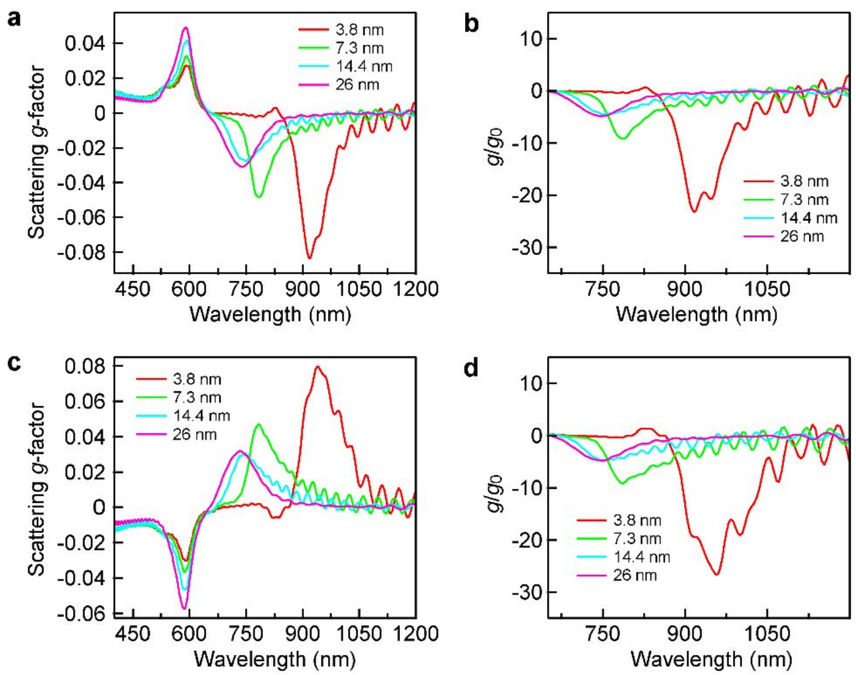

Figure S18. FDTD-simulated results for a CGNC supported on the Au/Al_2O_3 substrates under normal incidence of CPL. (a,b) Scattering *g*-factor and g/g_0 spectra of the D-handed CGNC on the Au/Al_2O_3 substrates with Al_2O_3 layer thicknesses of 3.8 nm (red lines), 7.3 nm (green lines), 14.4 nm (cyan lines), and 26 nm (pink lines). (c,d) Scattering *g*-factor and g/g_0 spectra of the L-handed CGNC on the Au/Al_2O_3 substrates.

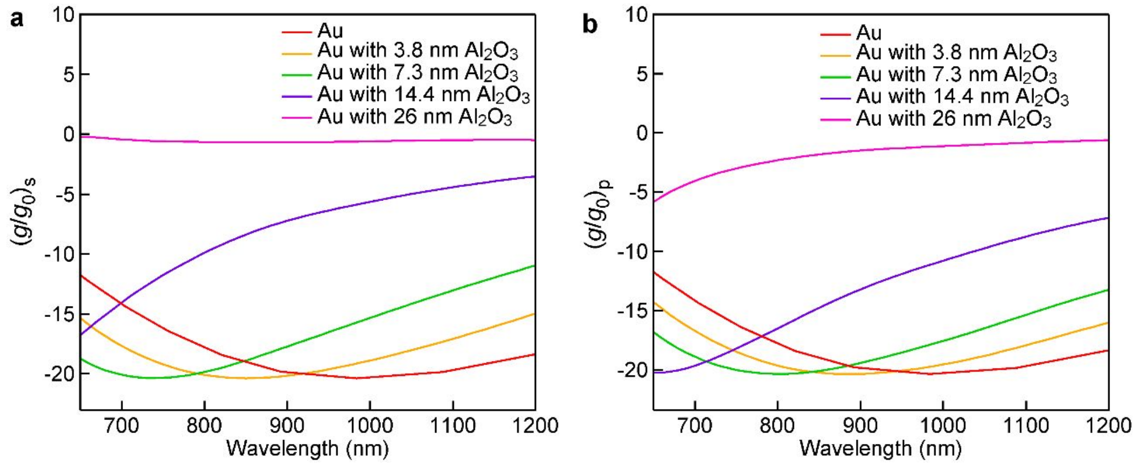

Figure S19. Spectra of $(g/g_0)_s$ and $(g/g_0)_p$ for the Au/Al_2O_3 bilayer substrates. (a) Spectra of $(g/g_0)_s$. The red, yellow, green, purple, and pink lines represent the $(g/g_0)_s$ spectra for the Au substrates and Au/Al_2O_3 substrates with Al_2O_3 layer thicknesses of 3.8, 7.3, 14.4, and 26.0 nm, respectively. (b) Spectra of $(g/g_0)_p$. The spectra of $(g/g_0)_s$ and $(g/g_0)_p$ were calculated under the excitation of the s- and p-polarized components of CPL at an oblique incidence angle of 64°, respectively. The minimum values of $(g/g_0)_s$ and $(g/g_0)_p$ in the wavelength range of 550–750 nm are increased as the thickness of the Al_2O_3 layer is increased from 3.8 nm to 14.4 nm.

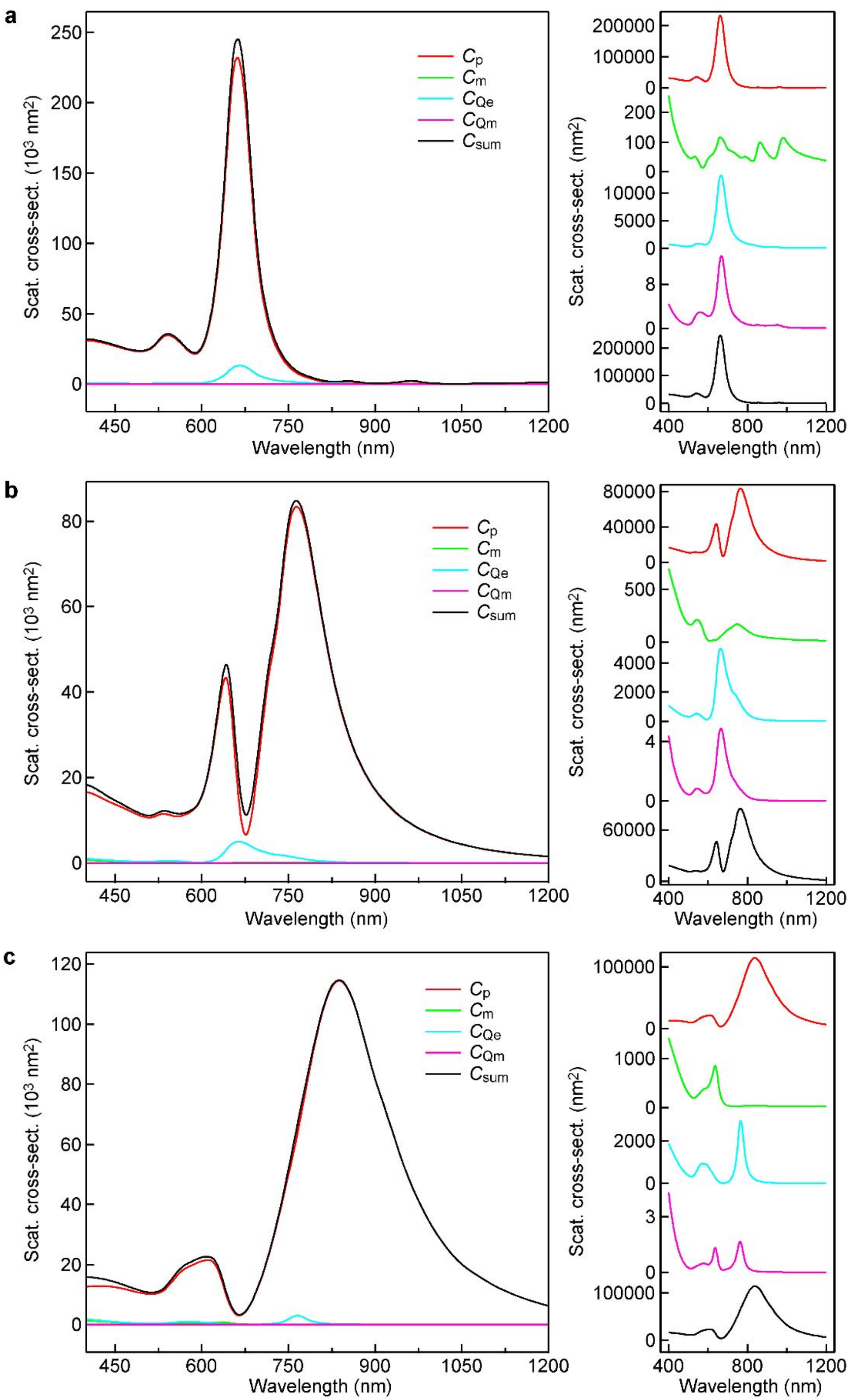

Figure S20. Multipolar expansion of the plasmon modes in a L-handed CGNC. (a) Scattering cross-section spectra of the electric dipole (ED), magnetic dipole (MD), electric quadrupole

(EQ), and magnetic quadrupole (MQ) modes, which are denoted by C_p, C_m, C_{Qe}, and C_{Qm}, respectively, in the CGNC on a gold substrate. (b) C_p, C_m, C_{Qe}, and C_{Qm} of the CGNC on a silica substrate. (c) C_p, C_m, C_{Qe}, and C_{Qm} of the CGNC in water. The black lines of C_{sum} represent the total scattering cross-section spectra by adding the C_p, C_m, C_{Qe}, and C_{Qm} together.

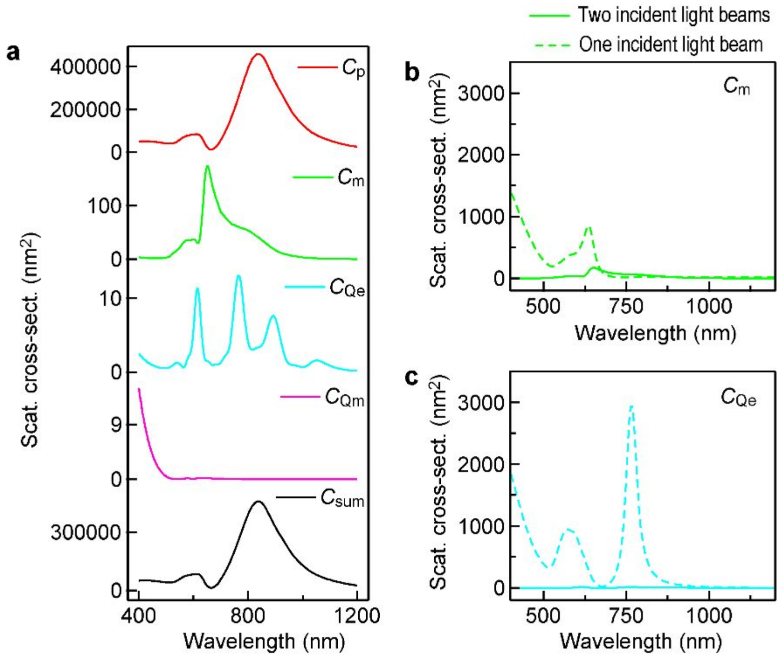

Figure S21. Multipolar expansion of the plasmon modes in a L-handed CGNC under the excitation of two opposite linearly polarized light beams. (a) Calculated C_p, C_m, C_{Qe}, and C_{Qm} of the CGNC in water. The black line of C_{sum} represents the total scattering cross-section spectrum by adding the C_p, C_m, C_{Qe}, and C_{Qm} together. (b) Comparison between the results of C_m under one normally incident linearly polarized light beam (dotted line) and two opposite linearly polarized light beams (solid line). (c) Comparison between the results of C_{Qe} under one normally incident linearly polarized light beam (dotted line) and two opposite linearly polarized light beams (solid line).
